# A Cationic-Independent Mannose 6-Phosphate Receptor Inhibitor (PXS64) Ameliorates Kidney Fibrosis by Inhibiting Activation of Transforming Growth Factor-β_1_

**DOI:** 10.1371/journal.pone.0116888

**Published:** 2015-02-06

**Authors:** Jie Zhang, Muh Geot Wong, May Wong, Simon Gross, Jason Chen, Carol Pollock, Sonia Saad

**Affiliations:** 1 Kolling Institute of Medical Research, Sydney, Australia; 2 Royal North Shore Hospital, St. Leonards, Australia; Faculty of Medicine & Health Sciences, UNITED ARAB EMIRATES

## Abstract

The activity of transforming growth factor-β1 (TGF-β_1_) is regulated by its conversion from the latent to the active form. We have previously shown that the conversion is at least in part mediated by the cationic-independent mannose 6-phosphate receptor (CI-M6PR), as the CI-M6PR inhibitor, PXS-25 has anti-fibrotic properties in human kidney tubular (HK-2) cells under high glucose conditions. However, its clinical use is limited by low bioavailability. Our aim was to determine the effects of PXS64, a pro-drug of PXS25, in *in vitro* and *in vivo* models of renal fibrosis. HK-2 cells were exposed to latent TGFβ_1_+/- PXS64 for 48 hours. The mRNA and protein levels of pro-fibrotic and pro-inflammatory markers were determined. A 7 day unilateral ureteric obstruction (UUO) model was used and the following experimental groups were studied: (i) Sham operated, (ii) UUO, (iii) UUO + telmisartan (iv) UUO + PSX64. HK-2 cells exposed to PXS64 reduced TGFβ mediated effects on collagen IV, fibronectin, macrophage chemotactic protein-1 (MCP-1) and phospho-smad2 protein expression, consistent with inhibition of the conversion of latent to active TGF-β_1_. PXS 64 treated UUO mice had a lower tubulointerstitial fibrosis index, collagen IV and fibronectin protein and mRNA expression when compared to untreated UUO mice. In addition, these animals had lower MCP-1 mRNA expression, reduced inflammarory cell infiltrate, as indicated by fewer CD45, F4/80 positive cells, and reduced phospho-Smad2 protein expression when compared to untreated UUO animals. Our data demonstrates that PSX64 is an effective anti-fibrotic agent by inhibiting the activation of latent TGF-β_1_.

## Introduction

Tubulointerstitial fibrosis is generally recognized as the final common pathway of chronic kidney disease (CKD) [1]. It is characterized by the accumulation of extracellular matrix (ECM), primarily consisting of collagen IV and fibronectin, which is considered a reliable prognostic indicator in CKD [2]. Convincing evidence from animals and clinical studies have clearly demonstrated that inhibiting the renin-angiotensin-aldosterone system (RAAS) pathways is effective in delaying progressive kidney disease [3]. However, progressive loss of residual kidney function occurs despite the use of RAAS inhibitors, suggesting that there is a need to discover novel non-RAAS therapeutics for the prevention of tubulointerstitial fibrosis.

Transforming growth factor-β1 (TGF-β_1_) is a multifunctional cytokine which plays a pivotal role in kidney fibrosis in addition to its multiple other roles such as in tissue regeneration, cell differentiation, apoptosis, adhesion and the regulation of the immune system[4]. It is commonly observed that tissue TGF-β_1_ expression is closely associated with aggregation of CD45+ leukocytes and F4/80+ macrophage infiltration, with increased pro-inflammatory mediators such as monocyte chemoattractant protein-1 (MCP1)[5]. TGF-β_1_ exists in abundance in its latent form bound to the Latency Associated Protein (LAP). Dissociation of TGF-β_1_ from the LAP results in the release of the biological active form. Active TGF-β_1_ interacts with its specific transmembrane TGFβ Type I (TβRI) and type II (TβRII) receptors and activates a cascade of intracellular signaling pathways, including the well-studied small mother against decapentaplegic (Smad), and others pathways such as protein kinase B (AKT) and the extracellular signal-regulated kinase (ERK) pathways[6–10]. Activation of latent to active TGF-β_1_ can be achieved *in vitro* by extreme heat and pH [11], urea[12], and several physiological activators including angiotensin II, proteases, metalloproteinase [13]and thrombosponin-1[14]. Physiological activation of latent TGF-β_1_ is well described *in vivo* via the plasminogen/ plasmin proteolytic system[15], binding to the cationic independent mannose 6-phosphate receptor (CI-M6PR) and by direct binding to integrins. Complete inhibition of TGF-β_1_ is irrelevant as TGF-β_1_ knockout animals exhibit a lethal phenotype at birth and treatment with a pan-antibody against TGF-β_1_ results in severe side effects, including fulminant sepsis[16].

In recent years, the cationic independent mannose 6-phosphate receptor (CI-M6PR) has been increasingly recognized to play a role in maintaining cellular homeostasis, in addition to its conventional role as a lysosomal carrier protein[17]. The extracellular domain of the 300kDa transmembrane glycoprotein has several mannose 6-phosphate binding sites that can bind to the LAP domains of the latent TGF-β_1_, resulting in conformation change and release of its active component. Hence, the mannose-6-phosphate (M6P) can be considered as an inhibitor for TGFβ1 activation by competitive binding with the CIM6PR. Previous studies suggested that M6P can reduce TGF-β_1_-induced injury seen in tendon and nerve repair, autoimmune encephalomyelitis, peritonitis and arthritis[18–21]. Based on this, analogues of M6P that selectively bind to the CI-M6PR have been developed as novel anti-fibrotic agents. We have previously shown that a selective CI-M6PR inhibitor, PXS25, can effectively prevent the activation of TGF-β_1_ and suppress the extracellular matrix expression in human kidney proximal tubular (HK2) cells under high glucose but not hypoxic conditions[22]. A lipophilic inhibitor, PXS64 was subsequently designed to improve its physiochemical properties. PXS64 is rapidly cleaved to PXS25 which potentially increases its absorption through the gastrointestinal tract. We aimed to determine the effectiveness and safety of PXS64 in the prevention of kidney fibrosis by using *in vitro* and *in vivo* models and the signaling pathways of PXS64-mediated renoprotection.

## Materials and Methods

### 1. Cell culture

HK2 cell lines were obtained from American Type Cell Collection (ATCC). Cells (passages 6–17) were maintained in six- well plates (Sarstedt, Germany) in Keratinocyte-Serum Free (KSF, Invitrogen 17005–075, USA) Medium cultured at 37°C in a humidified atmosphere of 5% CO_2_. These cells were exposed to 100ng/ml latent TGFβ1 (R&D Systems 299-LT-005) with or without 10 μM PXS64 (Courtesy of Pharmaxis ltd) for 48 hours. PXS64 was dissolved in DMSO and diluted in PBS buffer. Plain KSF medium (containing 5 mM D-glucose) plus the same amount of DMSO were used as the control. The supernatant and cell lysate were collected after 48 hours treatment.

### 2. In vivo studies

Six to eight-week-old male C57/6 mice weighing 20–25 g were used in this study. A total of 40 mice were divided into four groups: (i) 10 mice had left proximal unilateral ureteral ligation for 7 days as previously described [23, 24]. (UUO); (ii) 10 sham-operated mice as a control; (iii) 10 UUO mice receiving 3 mg/kg of Telmisartan (Sigma, USA) diluted in Phosphate buffered saline (PBS), administered in drinking water 5 days prior and 7 days after UUO procedure; (iv) 10 UUO mice receiving 10μM PXS64 (courtesy of Pharmaxis Ltd) diluted in olive oil by intraperitoneal injection for 7 days after the UUO procedure. All mice were fed as a standard diet and subjected to 12 hours of daylight and 12 hours darkness. The study design obtained ethical approval from the Animal Care and Ethics Committee (ACEC) of Kolling Institute of Medical Research, University of Sydney.

The weight and blood pressure of the animals were recorded before the UUO procedure and at the end of the study (Day 7). Blood pressure was measured by using non-invasive tail vein cuff methodology (CODA Blood Pressure apparatus, Kent Scientific, Connecticut, USA). 24 hour urines were collected in metabolic cages before the animals were sacrificed. Blood was collected by cardiac puncture at the time of sacrifice. The plasma was separated by high speed centrifugation for 10 minutes at 4°C and snap frozen using liquid nitrogen and stored at -80°C. Urinary albumin and creatinine levels were assessed by the Murine Microalbuminura and Microcreatuninura ELISA kit (Exocell, Inc., Philadelphia, PA, USA). Half of the perfused left ligated kidney was snap frozen for mRNA and protein studies. Two third of the another half of the ligated kidney was fixed in 10% buffered formalin solution overnight and stored into 70% ethanol before embedding in paraffin blocks. One third of the remaining left kidney was snap frozen in inert support medium such as optimal cutting temperature (OCT) OCT solution (Sakura Finetek USA) for frozen sections. The right kidneys were similarly processed as internal control.

### 3. Tubulointerstitial index

Paraffin embedded sections (5µm) were used for Masson’s trichrome staining and examined by a light microscope (Olympus photomicroscope linked to a DFC 480 digital camera). Semiquantitative scoring of the tubulointerstitial index was performed by random selection of 10 non-overlapping images of the kidney cortex using the following scoring system: score 0 = normal; score1 = mild interstitial fibrosis (≤ 10%); score2 = moderate interstitial fibrosis (10–25%); score3 = severe interstitial fibrosis (25–75%); score4 = extremely severe interstitial fibrosis (≥ 75%). The average scores from two blinded examiners were used for final analysis. The characteristics features for tubulointerstitial fibrosis included tubular atrophy or dilatation, presence of mononuclear inflammatory cells, widening of interstitial spaces with deposition of extracellular matrix, and interstitial cell proliferation.

### 4. Immunohistochemistry and semiquantitation

**A) Paraffin embedded sections**. For specific antibody detection, sections (5µm) were dewaxed in xylene and rehydrated in graded concentrations of ethanol. Antigen retrieval was firstly achieved by incubating the sections in 99°C citrate buffer (pH6.0) for 30 minutes, followed by washing in tap running water, then blocked in 10% blocking solution(Dako, Glostrup, Denmark) for 15 minutes. Thereafter, sections were incubated with primary antibodies against fibronectin (dilution 1:1000) (Sigma-Aldrich, Dublin, Ireland) and collagen IV (dilution 1:1000) (Abcam, Cambridge, UK) or the leukocyte common antigen CD45 (dilution 1:100) (Merck Millipore, Darmstadt, Germany) at 4°C overnight in a humid chamber and washed three times in buffered PBS before incubating with a secondary biotinylated antibody for 1 hour at room temperature (ABD Serotec, USA; dilution 1:200), and then treated with peroxidase-conjugated streptavidin for 15 minutes (Dako Glostrup, Denmark) to visualise the tissue immune complexes using LSAB+ detection system (Dako Glostrup, Denmark). Antigen-antibody reactions were visualized with chromogen diaminobenzidine and counter staining was performed using Mayer’s Haematoxylin followed by Scott’s Blue staining (Fronine, Taren Point, NSW, AU). Control sections were also prepared in which the authentic primary antibodies were replaced with an irrelevant isotype matched IgG.

**B) Frozen section**. Six-µm frozen sections were fixed in Acetone for 10 minutes immediately after removal from the freezer and did not undergo heat retrieval. Sections were then washed in PBS three times, 5 min each, pre-incubated in a blocking solution (10% goat serum in PBS) for 30 min and washed in PBS three times. They were then incubated in rat anti-mouse F4/80 monoclonal antibody (ABD Serotec, USA; dilution 1:100) and Rat anti-mouse CD68 antibody (ABD Serotec, USA; dilution 1:100) for one hour. Each section was washed three times in PBS and then incubated with a secondary HRP tagged goat anti rat antibody for 30 min (ABD Serotec, USA; dilution 1:200). Antigen-antibody reactions were then visualized with chromogen diaminobenzidine and counter staining was performed using Mayer’s Haematoxylin followed by Scott’s Blue staining (Fronine, Taren Point, NSW, AU).

**C) Semiquantitative analyses**. The tissue specimens were examined by bright field microscopy using an Olympus photomicroscope linked to a DFC 480 digital camera. For fibronectin, collagen IV, CD68, CD45 and F4/80, twenty consecutive non-overlapping fields from each section of renal cortex were photographed under high magnification (x400). Stained areas were quantified using a computer-aided manipulator (image-operating computer program, Image J (Java based software program, NIH). The percentage of the stained area relative to the whole area of the field in each visual field was determined.

### 5. Relative quantitative real-time RT-PCR

Total RNA was extracted from HK2 cells or fresh frozen ligated kidney tissue using an RNA isolation kit (RNeasy Mini Kit, Qiagen, Valencia, CA) according to the manufacturer’s instructions. Briefly, cDNA was synthesized by Transcriptor First Strand cDNA Synthesis Kit (Roche # 04897030001, Penzberg, Germany). Quantitative real-time PCR was performed to assess the expression of fibronectin, collagen IV, TGFβ1 and MCP1 (Table 1) by using ABI Prism 7900 HT Sequence Detection System (Applied Biosystems, Foster City, CA) with SYBR Green PCR Master Mix(Life Technologies # 4334973, USA). Data were analyzed by relative quantitation using RQ Manager Software, version 1.2 (Applied Biosystems, Foster City, CA) and presented as fold-change compared with control after normalization to β-actin.

**Table 1:** Details of primers used for pro-fibrotic and pro-inflammatory markers.

**Gene**	**Primer sequence**
mouse Fibronectin	Forward: 5’-CACGGAGGCCACCATTACT-3’
	Reverse: 5’-CTTCAGGGCAATGACGTAGAT-3’
mouse Collagen IV	Forward: 5’-TTAAAGGACTCCAGGGACCAC-3’
	Reverse: 5’-CCCACTGAGCCCTGTCACAC-3’
mouse TGF-β1	Forward: 5’-TCAGACATTCGGGAAGCAGT-3’
	Reverse: 5’- ACGCCAGGAATTGTTGCTAT -3’
mouse MCP-1	Forward: 5’-CATCCACGTGTTGGCTCA-3’
	Reverse:5’-GATCATCTTGCTGGTGAATGAGT-3’
mouse β-actin	Forward: 5’-CCCACTGAGCCCTGTCACAC-3’
	Reverse: 5’-GTGGTACGACCAGAGGCATAC-3’
human Fibronectin	Forward: 5’-GCGAGAGTGCCCCTACTCA-3’
	Reverse: 5’-GTTGGTGAATCGCAGGTCA-3’
human Collagen IV	Forward: 5’-CGGGTACCCAGGACTCATAG-3’
	Reverse: 5’-GGACCTGCTTCACCCTTTTC-3’
human β-actin	Forward: 5’-ATCGTGCGTGACATTAAG-3’
	Reverse: 5’-ATTGCCAATGGTGATGAC-3’
human MCP-1	Forward: 5’-CCAAAGAAGCTGTGATCTTCAA-3’
	Reverse: 5’-TGGAATCCTGAACCCACTTC-3’

### 6. Western blot analysis

Cells were scraped from culture plates and lysed in cold cell lysis buffer (containing protease and phosphate inhibitors in buffered PBS) and kidney tissues were homogenized in cold HES buffer including 20mM HEPES, 1mM EDTA, 250mM Sucrose and protease and phosphate inhibitors. The suspended solutions were vortexed for 5 minutes before centrifugation at 12,000 rpm, 4°C for 10 minutes. Total cell protein was assessed by Bio-Rad protein assay kit (Bio-Rad #500–0001, Bio-Rad Laboratories Ltd. Hertfordshire, UK) **according to manufacturer’s instructions**. 25 μg of total cell protein was analyzed by 4–12% sodium disulfide polyacrylamide gradients gel and electro-blotted to Hybond nitrocellulose membranes (Amersham Pharmacia Biotech, Bucks, UK). Membranes were incubated with primary antibodies against fibronectin (dilution 1:1000 Sigma-Aldrich, Dublin, Ireland), collagen IV (dilution 1:1000 Abcam, Cambridge, UK), phosphorylated Smad2 (dilution1:1000 Cell signaling Technology, Inc.), phosphorylated ERK1/2 (dilution 1:1000 Cell signaling Technology, Inc.), phosphorylated AKT (dilution 1:1000 Cell signaling Technology, Inc.), Smad2 (dilution 1:1000 Cell signaling Technology, Inc.), ERK1/2 (dilution 1:1000 Cell signaling Technology, Inc.), AKT (dilution 1:1000 Cell signaling Technology, Inc.) and actin (dilution 1:2000 Santa Cruz) overnight at 4°C after blocking with 5% skim milk for 1 hour at room temperature. All membranes were incubated with the appropriate horseradish peroxidase-conjugated secondary antibodies after washing in Tris Buffered Saline (TBS) with 5% Tween 20 three times. Proteins were visualized using the enhanced chemiluminescence (ECL) detection system (Amersham Pharmacia Biotech) and images were captured using LAS 4000 (Fujifilm, Tokyo, Japan). Images were exported by the Multigauge system (Fujifilm) and analyzed using Image J 1.47 software.

### 7. TGFβ1 and MCP1 immunoassays

The supernatant was collected at the time of cell harvesting at 48 h and concentrated by Amicon Ultra-4 Centrifugal Filter Units (Millipore, Bedford, MA, USA) by centrifugation at 4000 rpm until the volume was reduced to 500μl volume before storing at −80°C. Active TGFβ_1_ and MCP1 levels were detected by immunoassay (Promega, Madison, WI) as per the manufacturer’s instructions. The absorbance readings at 450 nm were read using a 96-well microplate reader. Levels were corrected for total cellular protein content assessed by Bio-Rad Protein assay (Bio-Rad Laboratories Ltd. Hertfordshire, UK). For tissue lysates, active TGFβ_1_ was determined using the same method using 100–300 µg of tissue.

### 8. Quantification of collagen release

Collagen content was measured in the tissue using hydroxy-proline as previously described [25]. Briefly, tissues were weighed, dried and then digested with papain overnight at 60^o^C. Digested tissues were then hydrolyzed in 6M HCl for 16 hrs at 110^o^C followed by neutralizing with 6M NaOH. The free hydroxyl-proline was then oxidized to pyrrole with chloramine T and the red color developed by the addition of p-dimethylaminobenzaldehyde (DMAB) and measured in a plate reader at 562nm. Data is expressed as hydroxyl-proline release (µg/mg of wet tissue).

### 9. Statistical analysis

Statview, version 4.5 (Anacus Concepts, Berkeley, CA) was used for all statistical analyses with p value <0.05 considered significant. All data are reported as mean ± SEM. Differences between groups were determined using ANOVA tests with posthoc Bonferroni tests. Western blot and real-time PCR data were presented as fold-change compared with the control value.

## Results

### 1. PXS64 inhibits activation of latent TGF-β_1_ in HK2 cells

**Figure 1:**
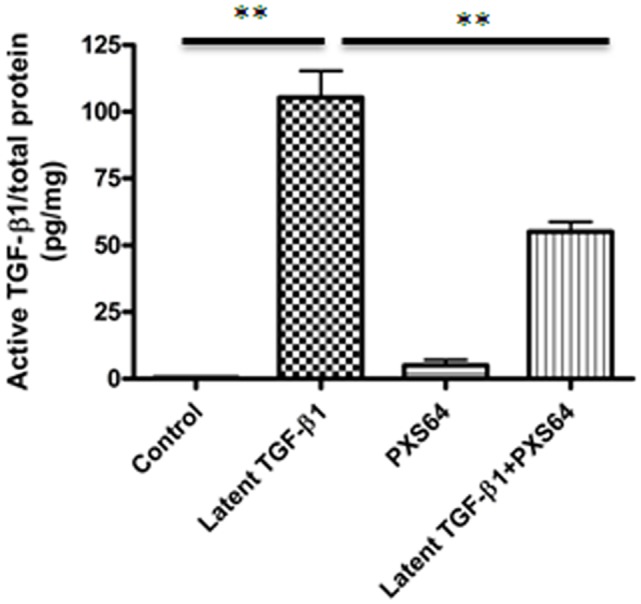
PXS64 suppressed conversion of latent to active TGFβ1 in HK2 cells. Active transforming growth factor TGF-β1 was determined in the concentrated supernatant after 48 hours exposure to 100ng/ml latent TGF-β1 with or without 10μM PXS64. There was increased active TGF-β1 concentration when cells were exposed to excess latent TGF-β1, which was markedly suppressed on co-exposure with PXS64. This data suggest that conversion of latent to active TGF-β1 can be effectively inhibited by a cationic independent mannose 6-phosphate receptor inhibitor (**P < 0.01 vs. control). Results are expressed as mean ± SEM, n = 3.

To establish the optimal concentration of PXS64, a dose response of PXS64 on HK2 cells was performed. PXS64 did not exhibit a proliferative or cytotoxic effect at the concentration of 1μM, 10μM and 20μM (data not shown). 10 μM was subsequently used for all *in vitro* experiments. When HK2 cells were exposed to 100ng/ml latent TGF-β_1_ for 48hours, there was a marked increase in active TGF-β_1_ production (105.4±10.01pg/mg, P<0.01) compared to control (0.5±0.12pg/mg). PXS64 alone had a negligible effect on endogenous active TGF-β_1_ production but co-incubation of PXS64 with latent TGF-β_1_ significantly reduced active TGF-β1 to (55 ±3.74pg/mg) (*P* < 0.01 vs latent TGF-β_1_ alone) (Fig. 1).

### 2. PXS64 suppressed mRNA and protein expression of pro-fibrotic and pro-inflammatory markers in HK2 cells

**Figure 2:**
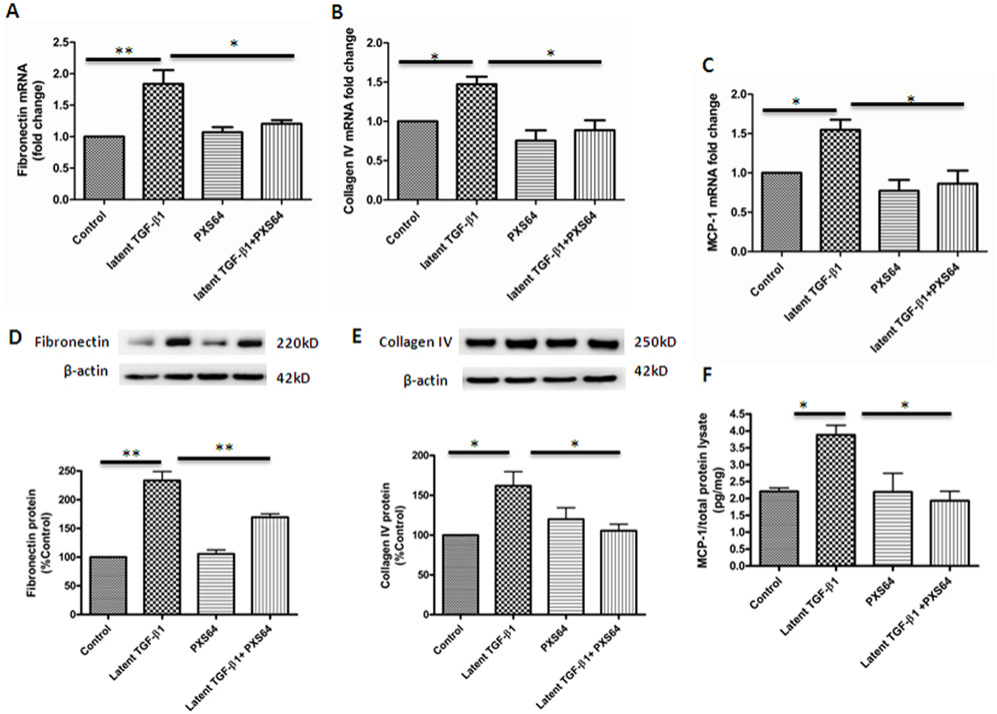
PXS64 inhibited TGF-β1-induced fibrotic and inflammatory markers in HK2 cells. HK2 cells exposed to 100ng/ml latent TGF-β1 showed a significant increase in fibronectin and collagen IV mRNA (Fig. 2A and B) and protein (Fig. 2D and E) expression, which were suppressed when co-exposed to PXS-64. These cells had increased mRNA expression of MCP-1 (Fig. 2C), which was translated to increased secretory MCP-1 in the supernatant (Fig. 2F). There were no difference mRNA and protein expression of the fibrotic and inflammatory markers s in HK2 cells under basal conditions or when cells were exposed to PXS64 alone (*P < 0.05, ** p<0.01 vs. appropriate control) Results are expressed as mean ± SEM, n = 4

TGF-β_1_ exposure resulted in increased fibronectin, collagen IV and MCP-1 mRNA levels to 1.8±0.22 fold P<0.01; 1.47± 0.10 fold and 1.5± 0.16 fold respectively (P<0.05 vs. control). The mRNA levels of fibronectin, collagen IV and MCP-1 were significantly reduced in the presence of PXS64 to (1.2 ±0.03; 0.89 ±0.11 and 0.9 ±0.17 fold respectively; P<0.05) (Fig. 2A, 2B and 2C). Similarly, HK2 cells treated with latent TGFβ_1_significantly induced fibronectin, collagen IV and MCP1 protein expression to 233±15%, P<0.01; 160± 18% and 3.94± 0.22pg/mg respectively (P<0.05 vs. control). Protein levels of fibronectin, collagen IV and MCP-1 were significantly reduced in the presence of PXS64 to 169 ±5.8% (P<0.01); 110 ±8% and 1.93 ±0.28pg/mg respectively (P<0.05 vs. latent TGF β_1_) (Fig. 2D, 2E and 2F). PXS64 alone had no effect on fibronectin, collagen IV and MCP-1mRNA and protein expression.

### 3. PXS64 selectively suppresses TGF-β_1_-Smad2 signaling pathway in HK2 cells

**Figure 3:**
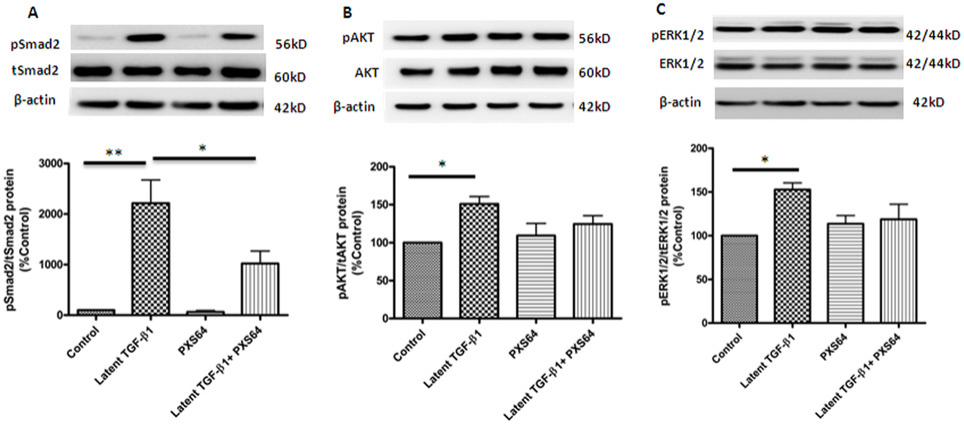
PXS64 suppressed TGF-β1-Smad signaling pathways in HK2 cells. Phosphorylated Smad2 expression was markedly increased when HK2 cells were exposed to 100ng/ml latent TGF-β1 for 48 hours. This was suppressed when PXS64 was added to the media, which strongly suggests that PXS64 suppresses TGF-β1-Smad2 rather than AKT or ERK signaling pathways (Fig. 3A, B and C). Results are expressed as mean ± SEM (n = 3, *P < 0.05, ** p<0.01 vs. appropriate control).

HK2 cells exposure to 100ng/ml of latent TGF-β_1_ markedly increased pSmad2 protein expression to 2214±458% (P< 0.01 vs. control). PXS64 significantly reduced TGF-β_1_ induced expression of pSmad2 to 1023±246% (P<0.05 vs. latent TGF-β_1_ effect) (Fig. 3A). Phosphorylated AKT (pAKT) and phosphorylated ERK (pERK) were high in HK2 cells at basal levels and their levels were significantly increased in TGF-β_1_ treated conditions to 144± 13% and 151± 9% respectively (P<0.05 vs. control). Interestingly, PXS64 treatment had no significant effect on pAKT and pERK protein expression 125±11% and 119 ±17% respectively, (both P>0.05 vs. latent TGF-β_1_) (Fig. 3B and 3C). These data suggest that the inhibitory effect of PXS64 is through a TGF-β_1_-Smad dependent signaling pathway.

### 4. Animal characteristics in the murine UUO model

There were 10 mice in each group. Most animals tolerated the intraperitoneal injection well. 3 animals died presumably due to peritonitis. Table 2 confirmed there was no difference in the weight, mean arterial blood pressure and urinary albumin creatinine ratio between all groups of UUO animals.

**Table 2:** Metabolic and physiological parameters of UUO mice treated with Telmisartan or PXS64.

**Each group (n = 8–12)**	**Sham**	**UUO**	**UUO+Telmisartan**	**UUO+PXS64**
Weight (g)	21.29±0.84	19.53±0.63	21.48±0.92	20.55±0.56
Final systolic blood pressure (mmHg)	98.19±6.17	102.63±2.43	97.73±4.49	98.01±2.94
Final mean blood pressure (mmHg)	81.41±5.49	83±2.45	80.78±3.52	80.67±3.46
Urinary albumin creatinine ratio (mg/mmol)	0.4±0.1	0.6±0.3	0.7±0.1	0.6±0.1

### 5. PXS64 reduced unilateral ureteral obstruction induced tubulointerstitial fibrosis

**Figure 4:**
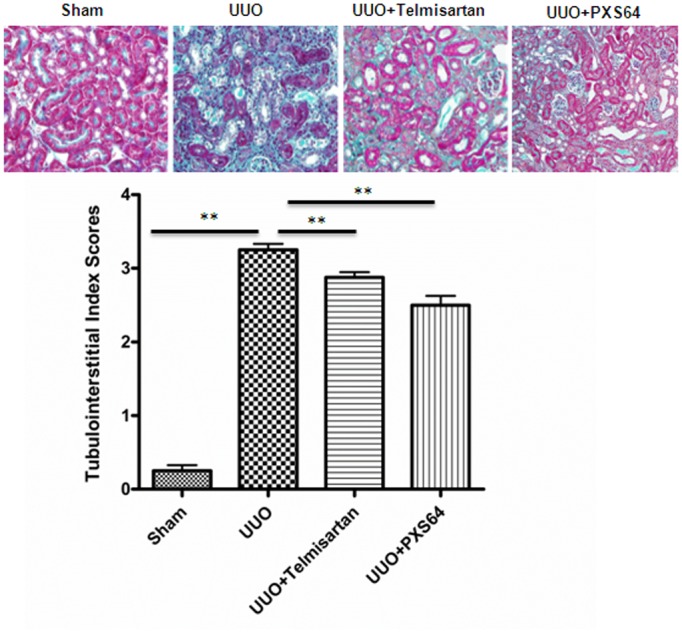
Animals treated with PXS64 showed reduced tubulointerstitial fibrosis in the unilateral ureteric obstruction (UUO) model. There was a markedly increased tubulointerstitial fibrosis index in animals that had undergone unilateral ureteric ligation (UUO) when compared to sham operated control (Sham) animals. Pre-treatment with telmisartan (UUO +telmisartan) or administration of PXS64 (UUO+PXS64) on the same day of UUO procedure, significantly reduced the tubulointerstitial fibrosis score. Results are presented as mean ± SEM. ** P < 0.01. Magnification x 400.

**Figure 5:**
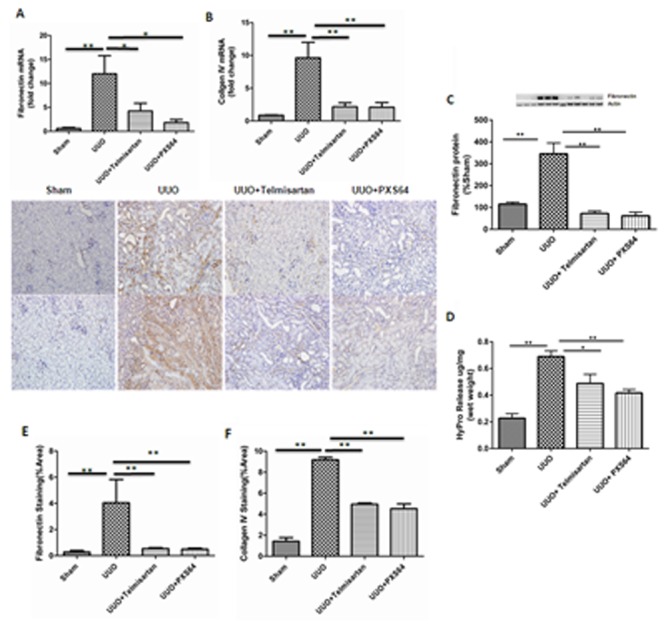
PXS64 reduced extracellular matrix mRNA and protein expressions in the UUO. Real time PCR showing increased fibronectin and collagen IV mRNA levels in UUO mice vs. sham and reduced levels by telmisartan and PXS64 (Fig. 5A and B). Western blot and Hydroxy-proline showing fibronectin protein expression and collagen release in the UUO kidney, telmisartan and PXS64 treated mice (Fig. 5C and Fig. 5D respectively). Western blot shows three lanes of each group representing three independent experiments of sham, UUO control, UUO+telmisartan and UUO+PXS64. Immunostaining of kidney tissue showing fibronectin and collagen IV protein expression in UUO mice in the absence and presence of telimisartan or PXS64 (Fig. 5E and 5F). Isotype specific IgG1 was used to confirm specificity of the staining (data not shown). (n = 8, *P < 0.05, ** P < 0.01).

The renal histology from UUO mice demonstrated increased Masson Trichrome staining and a higher tubulointerstitial index compared to control (3.2± 0.16 of UUO vs. 0.2 ±0.13 of sham, P<0.01). Animals treated with both telmisartan and PXS64 showed lower tubulointerstitial index scores and reduced histological evidence of tubular injury to 2.8 ±0.13 and 2.5 ±0.22 respectively P<0.01 vs. UUO (Fig. 4). UUO mice expressed increased levels of fibronectin (12± 3.79 fold vs. 0.6 ±0.19 fold of sham, P<0.01) and collagen IV mRNA levels (9.6± 2.38 vs. 0.8 ±0.05 fold of sham, P<0.01). PXS64 treatment significantly reduced fibronectin and collagen IV mRNA expression to (1.8±0.64 fold, P<0.05 vs UUO and 2.1±0.71 fold, P<0.01 vs. UUO respectively. The effect of PXS64 was similar to telmisartan’s effect, which significantly reduced fibronectin and collagen IV mRNA expression to (4.2±1.6 fold, p<0.05 and 2.2±0.6 fold respectively, P<0.01 vs. UUO) (Fig. 5A and 5B). Similarly, fibronectin protein expression was significantly increased in the UUO mice compared to sham (314.6±37.9% vs. 107.5± 8%; P<0.01 vs. sham). Both telmisartan and PXS64 significantly reduced fibronectin expression to (92.5±15% and 71.9±13.4%; P<0.01 vs. UUO respectively) (Fig. 5C). Collagen content was also significantly increased in UUO mice compared to sham (0.69 ±0.04 µg/mg vs. 0.23 ± 0.03 µg/mg; P<0.01). Telmisartan and PXS64 significantly reduced collagen content to 0.48 ±0.06 µg/mg; P<0.05 and 0.4 ±0.02 µg/mg; P<0.01 vs. UUO respectively (Fig. 5D). In addition, fibronectin and collagen IV protein expression were significantly increased in the UUO mice (fibronectin 5.5± 0.53 vs. 0.4±0.05%/area and collagen IV, 9.2± 0.44 vs. 1.4 ±0.68%/area, P<0.01), which was significantly reduced when animals were treated with either telmisartan (0.5 ±0.11 and 4.9±0.22%/area respectively, P<0.01 vs. UUO) or PXS64 (0.5±0.18 and 4.5±0.78%/area respectively, P<0.01 vs. UUO) (Fig. 5E and 5F).

### 6. PXS64 inhibits interstitial neutrophil and macrophage infiltration

**Figure 6:**
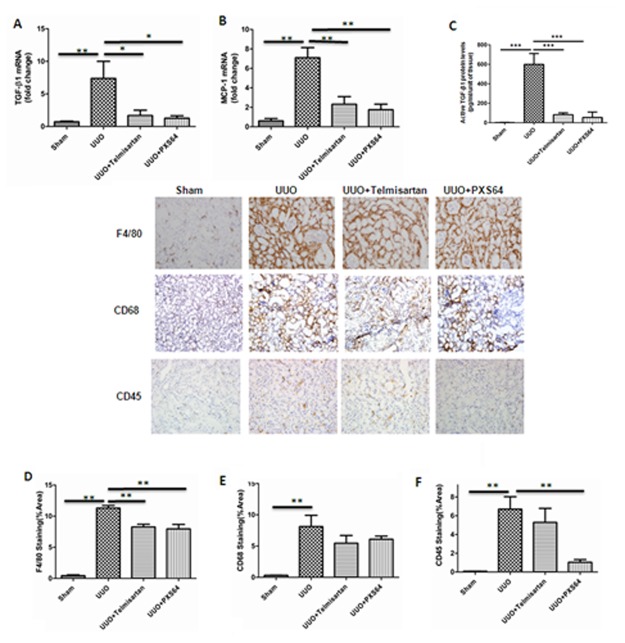
Reduction in cellular infiltration and inflammatory markers in UUO kidneys exposed to telmisartan or PXS64. Untreated UUO kidneys showed increased F4/80, CD68 and CD45 positively stained cells as compared to the sham operated control animals. PXS64 significantly reduced F4/80 and CD45 positive stained cells (Fig. 6C and E) with a trend to a reduction in CD68 cells, although this was not statistically significant (Fig. 6D). Telmisartan treated kidneys showed a reduction in F4/80 positive cells but no difference in CD45 or CD68 stained cells, suggesting a differential action of PXS64 and telmisartan in modifying cellular infiltration. Results are presented as mean showed (n = 8, *P < 0.05 vs. UUO, ** P < 0.01 vs. UUO). Magnification x 400.

UUO kidneys expressed increased TGF-β_1_ and MCP-1 mRNA levels (TGF-β_1_ 7.4±0.62 fold of UUO vs. 0.7±0.10 fold of sham; MCP-1, 7.1±0.45 fold of UUO vs. 0.6±0.24 fold of sham operated, p<0.01). Both levels were significantly reduced with PXS64 and telmisartan (Fig. 6A and 6B). Similarly active TGF-β_1_ was significantly increased in UUO mice to 599.4±111.4 pg/ml;P<0.001 vs. sham. Telmisartan and PXS64 significantly reduced active TGF-β_1_ protein levels to 83±18.8pg/ml and 55± 45pg/ml; P<0.001 vs. UUO (Fig. 6C). In addition, UUO mice had increased interstitial accumulation of CD45 (neutrophil marker) CD68 and F4/80 (macrophage markers) positive cells when compared to the sham operated animals (CD45, 6.7±2.3%/area; CD68, 8.1±3.15%/area and F4/80,11.3± 0.76%/area. P<0.01 vs. sham). Telmisartan significantly reduced F4/80 to (8.3± 0.79%/area, P<0.01vs UUO) but had no significant effect on CD68 and CD45 expression. However, PXS64 significantly reduced F4/80 to (7.9 ± 1.26%/area, P<0.01 vs. UUO) and had a marked reduction in the accumulation of CD45 positive cells as compared to the untreated UUO animals (1.1 ±0.49%/area, P<0.01 vs. UUO) (Fig. 6D, 6E and 6F). This suggests that the anti-inflammatory effect of PXS64 is via the suppression of interstitial neutrophil and macrophage infiltration.

### 7. PXS64 treated UUO kidneys had reduced phosphorylated Smad2 expression

**Figure 7:**
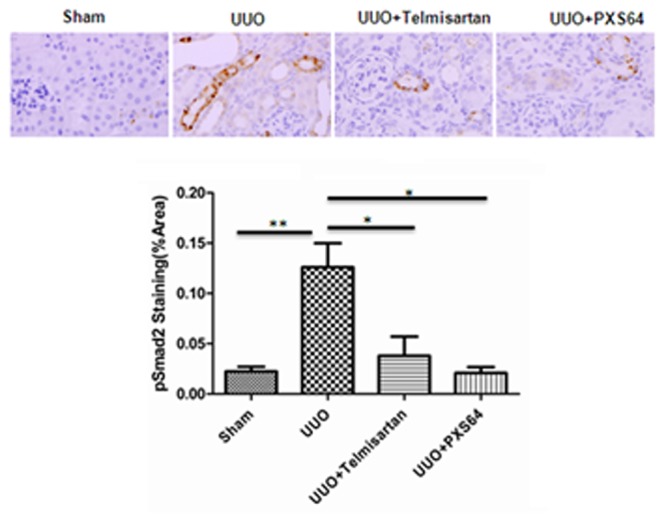
PXS64 reduced pSmad2 staining in UUO kidney cortex. Unilateral ureteric ligation increased tubular pSmad2 expression in the kidney cortex, which was markedly reduced when treated with PXS64 and telmisartan (Fig. 7). Results are presented as Mean +/-SEM. (n = 8).

Since our *in vitro* data demonstrated that PXS64 mediates its anti-fibrotic and anti-inflammatory effects via the TGF-β_1_/Smad2 dependent pathway, the level of pSmad2 protein expression was examined in the UUO model. The basal level of pSmad2 expression was very low. However, UUO kidneys increased tubular pSmad2 protein expression by 5 fold compared to the sham operated kidneys (p<0.01). Both telmisartan and PXS64 treated UUO kidneys reduced tubular pSmad2 expression by 60% and 80% respectively compared to the untreated animals (p<0.05) (Fig. 7). These data suggest that the anti-fibrotic and anti-inflammatory effects of PXS64 are via TGFβ1/Smad2 dependent pathway.

## Discussion

This study was undertaken to determine whether inhibition of the CI-M6PR may ameliorate renal tubulointerstitial fibrosis and inflammation in human proximal tubule cells and in an acute kidney fibrosis model. We subsequently aimed to elucidate the underlying mechanisms responsible for any renoprotection. Our data uniquely demonstrates that the M6P analogue, PXS64, a selective inhibitor of CI-M6PR, significantly attenuated inflammation and matrix protein expression in human proximal tubule cells and in a model of acute kidney fibrosis. In addition, we provide evidence that PXS64 exerts its effects in both *in vitro* and *in vivo* models via the conventional TGF-β_1_ Smad2-dependent pathway.

Tubulointerstitial fibrosis is widely accepted as a key pathophysiological change in all progressive renal diseases and a common final pathway leading to chronic kidney failure. During its progression, a number of biological events have been described, such as the release of cytokines, deposition of extracellular matrix, inflammatory cell infiltration, epithelial cell loss and tubular atrophy[26, 27]. It is well known that TGF-β_1_ signaling pathway can be activated by an acidic PH, exposure to high glucose, reactive oxygen species, proteases and metalloproteases[11, 13, 14, 22]. The activation of TGF-β_1_ can occur through binding to the CIM6PR or other *in vitro* pathways such as throbmospoin-1. Activated TGF-β_1_ can initiate a series of downstream signaling pathways in the cytoplasm, contribute to matrix protein deposition and inflammation and subsequently promote tubulointerstitial fibrosis. M6P is believed to block TGF-β_1_ induced tubulointerstitial fibrosis in flexor tendon injury, nerve injury repair and in autoimmune disease. Theoretically, M6P and its analogues may inhibit the binding of latent TGF-β_1_ to the CI-M6PR, thereby blocking the cleavage of the acid-labile subunit and the elaboration of active TGF-β_1_. In our study, we observed that PXS64 reduces active TGF-β_1_ production in HK2 cells suggesting that the CI-M6PR plays an important role in TGFβ1 activation in HK2 cells. The fact that PXS64 does not completely inhibit TGFβ1 activation is promising as complete inhibition of TGFβ would have a detrimental effect. We have additionally demonstrated that PXS64 suppresses the deposition of fibronectin and collagen IV in human proximal tubular cells exposed to latent TGF-β_1_ for 48hours. This finding is very consistent with a previous publication in our lab, suggesting PXS25 plays an inhibitory role in the expression of fibronectin and collagen IV induced by high glucose in human proximal tubular cells. In this study, we also investigated extracellular matrix production in a murine model of acute kidney fibrosis. These studies demonstrated very similar results to those observed in our *in vitro* studies22. Concurrent treatment with PXS64 inhibited mRNA and protein expression of fibronectin and collagen IV in a model of acute ureteric obstruction. During progression of tubulointerstitial fibrosis, the accumulation of leukocytes is an important step in mediating matrix protein production, fibroblast proliferation and tubular damage[28, 29]. We found that the UUO model leads to a higher expression of pro-inflammatory chemokines (MCP-1) and leukocyte accumulation (CD45) as well as macrophage infiltration (F4/80 and CD68). mRNA expression of MCP-1 and TGF-β_1_ were simultaneously upregulated in the UUO model group. Importantly, PXS64 inhibited MCP-1 expression and the infiltration of leukocytes and macrophages, suggesting the CI-M6PR inhibitor PXS64 may be beneficial in reversing the inflammatory process induced by UUO.

To further elucidate the mechanism by which PXS64 exerts anti-fibrotic and anti-inflammatory effects, we examined the effect of PXS64 on TGFβ1 induced Smad2, AKT and ERK1/2 activities in cultured human proximal tubular cells. Smad2 is an intracellular protein which mediates transduction of extracellular signals from TGFβ1 to activate nuclear transcription[30]. ,A number of anti-fibrotic agents, including Norcantharidin [31], Cordyceps sinensis[32], IL-7[33], proteasome inhibitor(MG132 and lactacystin) [34], mycophe[35] and PXS25 [22] have been shown to inhibit the TGFβ1-induced Smad2 pathway in both animal models of renal disease and in cultured tubular cells. We found that PXS64 (10µm) markedly suppresses the expression of pSmad2 in HK2 cells exposed to latent TGF-β_1_. Consequently, we examined another two TGF-β_1_ mediated signaling pathways (AKT and ERK1/2), but PXS 64 exerted no inhibitory effect on these pathways. Similarly, we detected upregulated pSmad2 expression in obstructed kidneys by immunohistochemical analysis, which was attenuated by PXS64 treatment. Although the levels of other Smad isoforms weren’t investigated in this study, the data suggest that the anti-fibrotic and anti-inflammatory effects of PXS64 are mediated by suppression of the TGF-β_1_/Smad pathway.

A limitation of this study is the lack of a chronic renal fibrosis model. The model of unilateral ureteral obstruction (UUO) can recapitulate fundamental pathophysiological mechanisms of chronic kidney diseases in a relatively short time span (7–14 days). It leads to many key features of fibrogenic responses, such as inflammatory cells influx, pro-fibrotic cytokines synthesis, matrix proteins production and tubular atrophy due to cell loss[36, 37]. However, in most clinical settings, kidney damage is a slowly progressive rather than acute process, and different signaling pathways may dominate with different causes of kidney disease.

## Conclusion

In summary, the current study is the first to demonstrate that PXS64, a selective CI-M6PR inhibitor can suppress production of extracellular matrix proteins, inhibit activation of the pro-fibrotic factor TGF-β_1_ and expression of pro-inflammatory cytokines through inhibition of the Smad2 signaling pathway. This study suggests a novel therapeutic approach for attenuating the progression of renal tubulointerstitial fibrosis.
