# Correction: A Cationic-Independent Mannose 6-Phosphate Receptor Inhibitor (PXS64) Ameliorates Kidney Fibrosis by Inhibiting Activation of Transforming Growth Factor-β1

**DOI:** 10.1371/journal.pone.0262725

**Published:** 2022-01-12

**Authors:** Jie Zhang, Muh Geot Wong, May Wong, Simon Gross, Jason Chen, Carol Pollock, Sonia Saad

After publication of this article [1], the authors notified *PLOS ONE* that the incorrect image was used in the UUO+PXS64 panel of Fig 4. In the updated version of Fig 4 provided with this notice, the erroneous panel has been replaced with the correct representative image from the original experiments. A member of *PLOS ONE*’s Editorial Board approved the updated figure and confirmed that the update does not alter the article’s conclusions.

**Fig 4:**
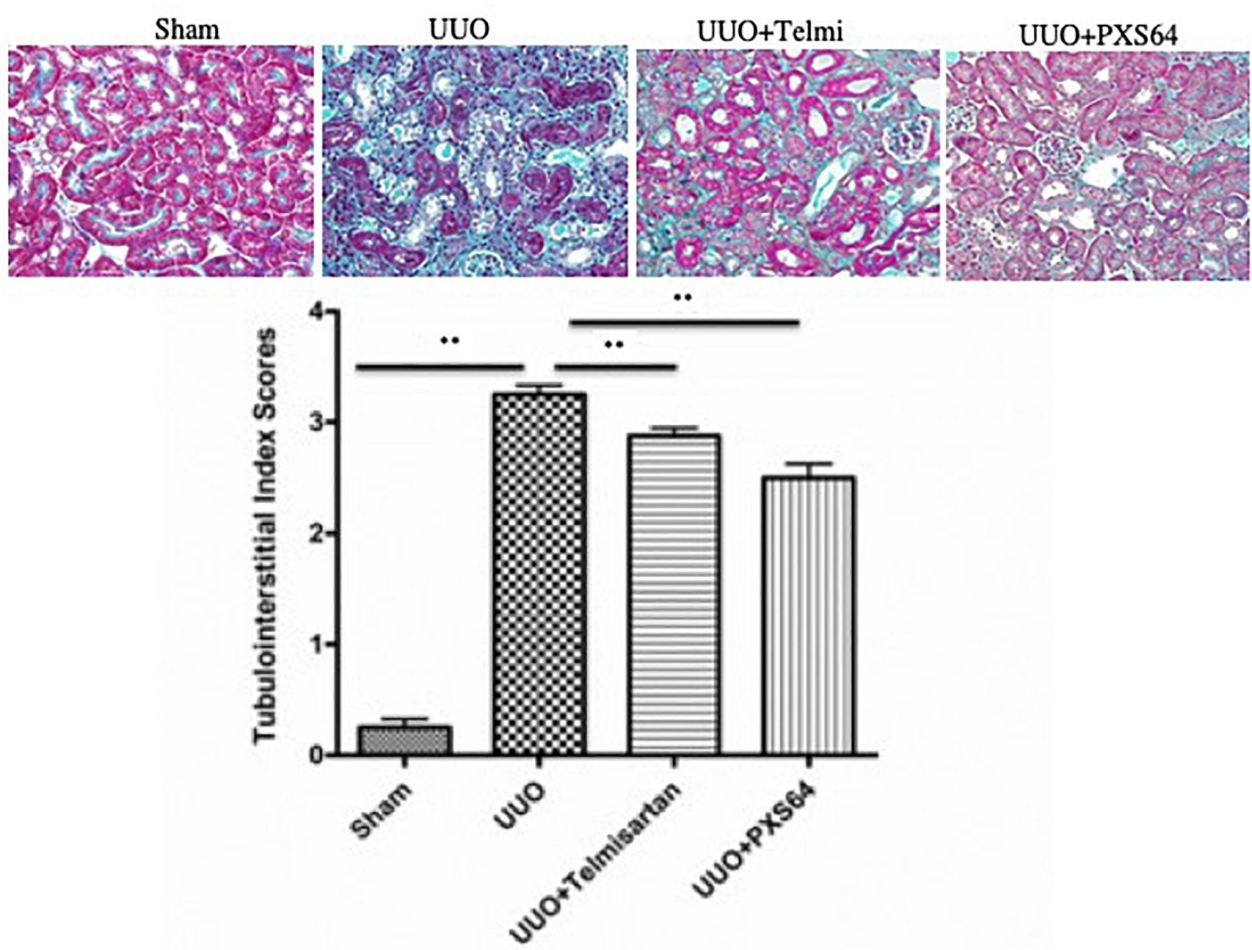
Animals treated with PXS64 showed reduced tubulointerstitial fibrosis in the unilateral ureteric obstruction (UUO) model. There was a markedly increased tubulointerstitial fibrosis index in animals that had undergone unilateral ureteric ligation (UUO) when compared to sham operated control (Sham) animals. Pre-treatment with telmisartan (UUO +telmisartan) or administration of PXS64 (UUO+PXS64) on the same day of UUO procedure significantly reduced the tubulointerstitial fibrosis score. Results are presented as mean ± SEM. ** P < 0.01. Magnification x 400.

The primary data underlying results in this article were not included with the original publication. The raw data underlying the article’s quantitative results are provided with this notice in S1–S3 Files.

## Supporting information

S1 File Quantitative data underlying graphs in Figs 1–3 and Figs 5–7. (PPTX)

S2 File Quantitative data underlying graph in Fig 4. (PDF)

S3 File Quantitative data underlying results reported in Table 2. (XLSX)
