# Supplementary material for: Correction: A Cationic-Independent Mannose 6-Phosphate Receptor Inhibitor (PXS64) Ameliorates Kidney Fibrosis by Inhibiting Activation of Transforming Growth Factor-β1
Source: PLoS One. 2022 Jan 12;17(1):e0262725. doi: 10.1371/journal.pone.0262725 (PMC8754332; doi:10.1371/journal.pone.0262725)
Supplement: S1 File — (PPTX) [file pone.0262725.s001.pptx]

## Slide 1
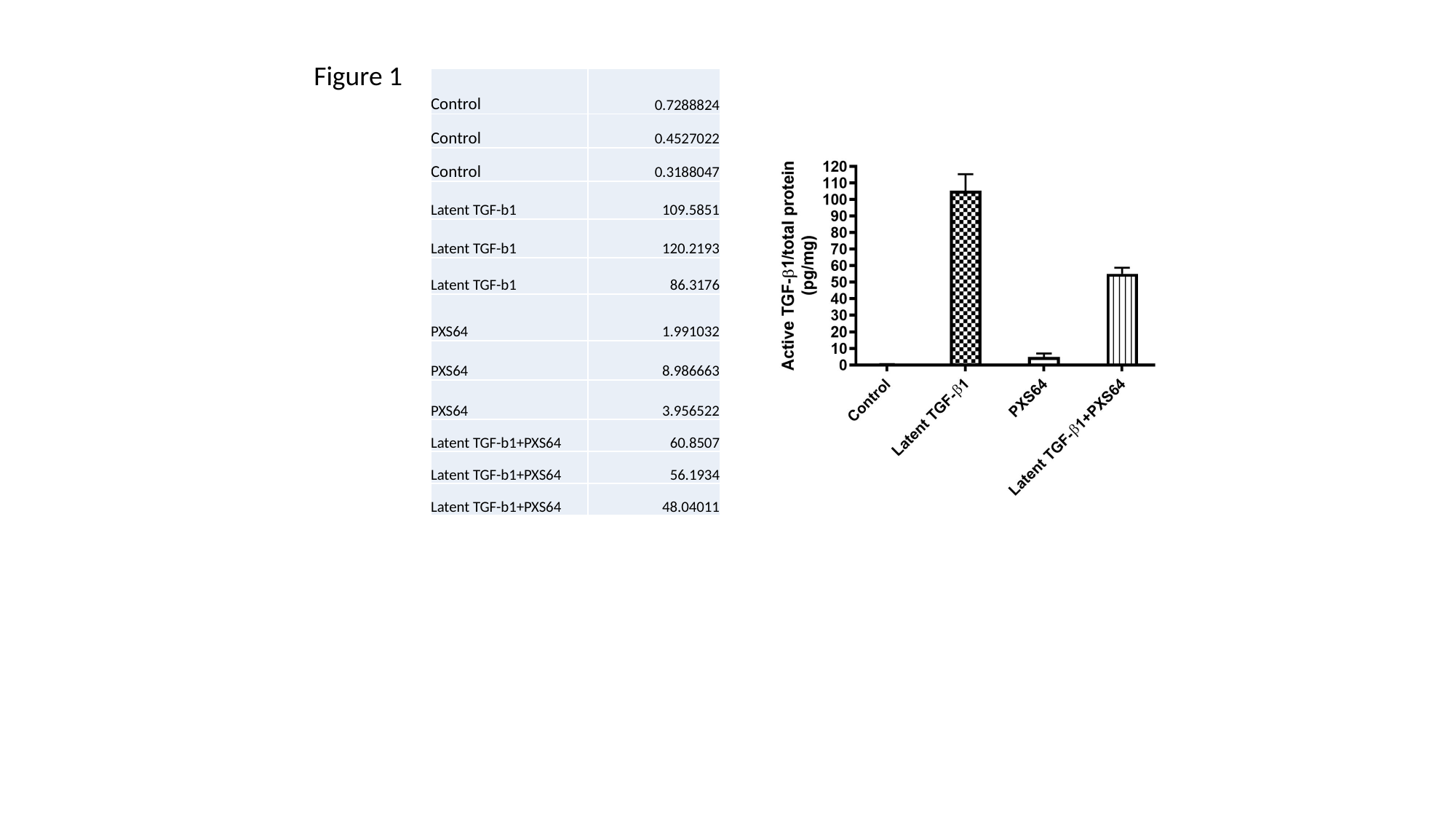

Figure 1
| Control | 0.7288824 |
| --- | --- |
| Control | 0.4527022 |
| Control | 0.3188047 |
| Latent TGF-b1 | 109.5851 |
| Latent TGF-b1 | 120.2193 |
| Latent TGF-b1 | 86.3176 |
| PXS64 | 1.991032 |
| PXS64 | 8.986663 |
| PXS64 | 3.956522 |
| Latent TGF-b1+PXS64 | 60.8507 |
| Latent TGF-b1+PXS64 | 56.1934 |
| Latent TGF-b1+PXS64 | 48.04011 |

## Slide 2
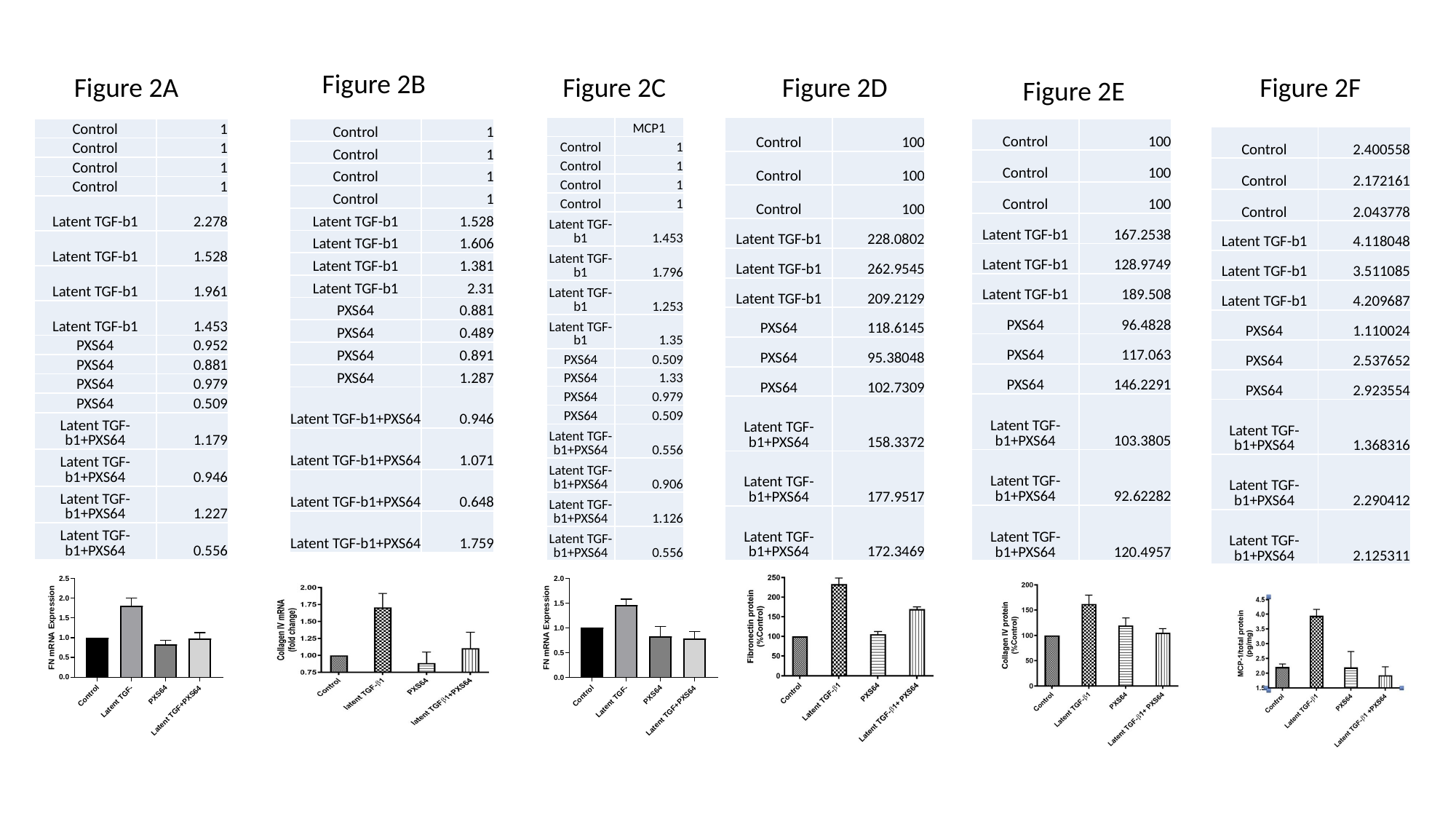

Figure 2B
Figure 2A
Figure 2C
Figure 2D
Figure 2F
Figure 2E
| | MCP1 |
| --- | --- |
| Control | 1 |
| Control | 1 |
| Control | 1 |
| Control | 1 |
| Latent TGF-b1 | 1.453 |
| Latent TGF-b1 | 1.796 |
| Latent TGF-b1 | 1.253 |
| Latent TGF-b1 | 1.35 |
| PXS64 | 0.509 |
| PXS64 | 1.33 |
| PXS64 | 0.979 |
| PXS64 | 0.509 |
| Latent TGF-b1+PXS64 | 0.556 |
| Latent TGF-b1+PXS64 | 0.906 |
| Latent TGF-b1+PXS64 | 1.126 |
| Latent TGF-b1+PXS64 | 0.556 |
| Control | 100 |
| --- | --- |
| Control | 100 |
| Control | 100 |
| Latent TGF-b1 | 228.0802 |
| Latent TGF-b1 | 262.9545 |
| Latent TGF-b1 | 209.2129 |
| PXS64 | 118.6145 |
| PXS64 | 95.38048 |
| PXS64 | 102.7309 |
| Latent TGF-b1+PXS64 | 158.3372 |
| Latent TGF-b1+PXS64 | 177.9517 |
| Latent TGF-b1+PXS64 | 172.3469 |
| Control | 1 |
| --- | --- |
| Control | 1 |
| Control | 1 |
| Control | 1 |
| Latent TGF-b1 | 2.278 |
| Latent TGF-b1 | 1.528 |
| Latent TGF-b1 | 1.961 |
| Latent TGF-b1 | 1.453 |
| PXS64 | 0.952 |
| PXS64 | 0.881 |
| PXS64 | 0.979 |
| PXS64 | 0.509 |
| Latent TGF-b1+PXS64 | 1.179 |
| Latent TGF-b1+PXS64 | 0.946 |
| Latent TGF-b1+PXS64 | 1.227 |
| Latent TGF-b1+PXS64 | 0.556 |
| Control | 1 |
| --- | --- |
| Control | 1 |
| Control | 1 |
| Control | 1 |
| Latent TGF-b1 | 1.528 |
| Latent TGF-b1 | 1.606 |
| Latent TGF-b1 | 1.381 |
| Latent TGF-b1 | 2.31 |
| PXS64 | 0.881 |
| PXS64 | 0.489 |
| PXS64 | 0.891 |
| PXS64 | 1.287 |
| Latent TGF-b1+PXS64 | 0.946 |
| Latent TGF-b1+PXS64 | 1.071 |
| Latent TGF-b1+PXS64 | 0.648 |
| Latent TGF-b1+PXS64 | 1.759 |
| Control | 100 |
| --- | --- |
| Control | 100 |
| Control | 100 |
| Latent TGF-b1 | 167.2538 |
| Latent TGF-b1 | 128.9749 |
| Latent TGF-b1 | 189.508 |
| PXS64 | 96.4828 |
| PXS64 | 117.063 |
| PXS64 | 146.2291 |
| Latent TGF-b1+PXS64 | 103.3805 |
| Latent TGF-b1+PXS64 | 92.62282 |
| Latent TGF-b1+PXS64 | 120.4957 |
| Control | 2.400558 |
| --- | --- |
| Control | 2.172161 |
| Control | 2.043778 |
| Latent TGF-b1 | 4.118048 |
| Latent TGF-b1 | 3.511085 |
| Latent TGF-b1 | 4.209687 |
| PXS64 | 1.110024 |
| PXS64 | 2.537652 |
| PXS64 | 2.923554 |
| Latent TGF-b1+PXS64 | 1.368316 |
| Latent TGF-b1+PXS64 | 2.290412 |
| Latent TGF-b1+PXS64 | 2.125311 |

## Slide 3
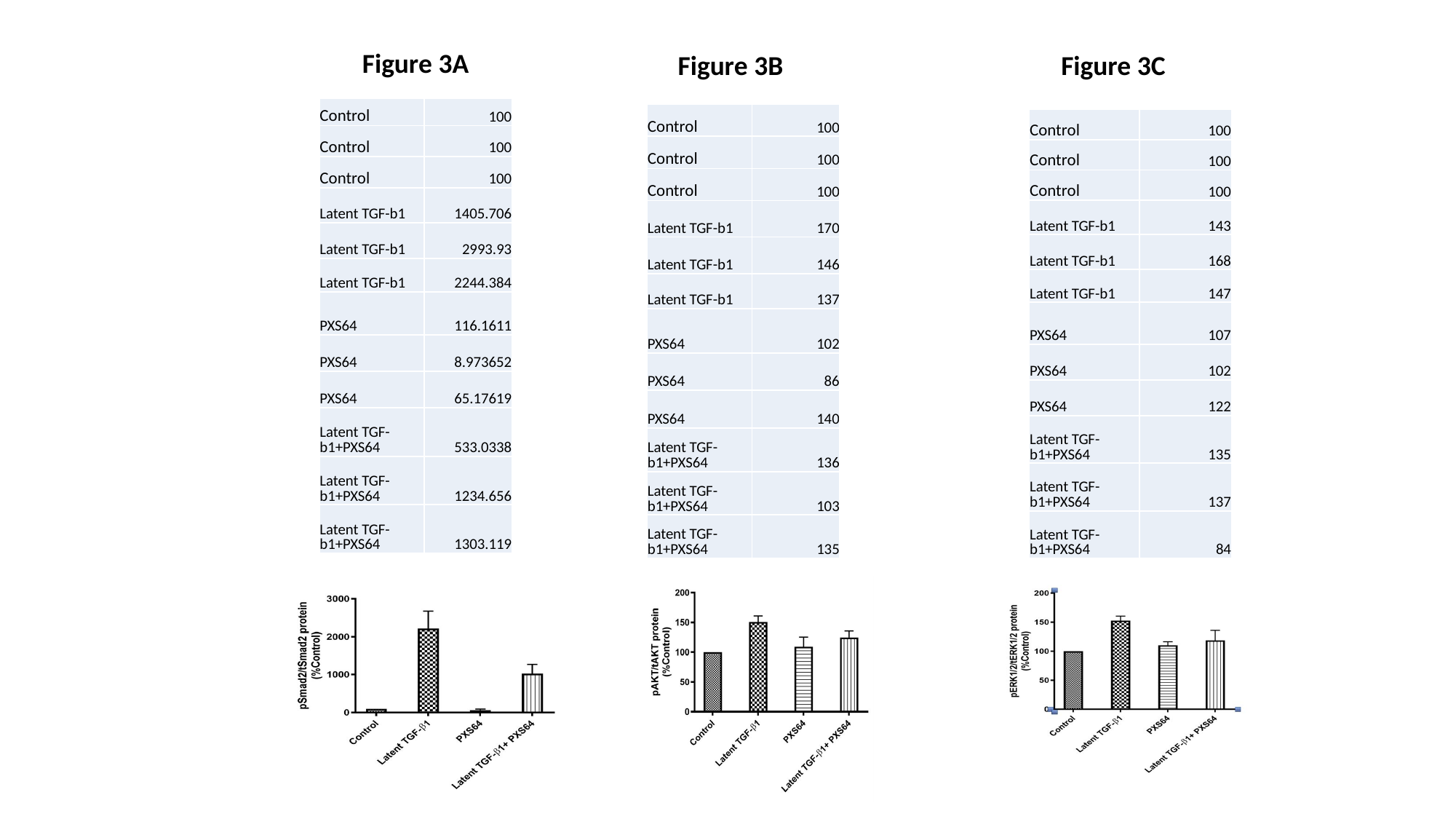

Figure 3A
Figure 3B
Figure 3C
| Control | 100 |
| --- | --- |
| Control | 100 |
| Control | 100 |
| Latent TGF-b1 | 1405.706 |
| Latent TGF-b1 | 2993.93 |
| Latent TGF-b1 | 2244.384 |
| PXS64 | 116.1611 |
| PXS64 | 8.973652 |
| PXS64 | 65.17619 |
| Latent TGF-b1+PXS64 | 533.0338 |
| Latent TGF-b1+PXS64 | 1234.656 |
| Latent TGF-b1+PXS64 | 1303.119 |
| Control | 100 |
| --- | --- |
| Control | 100 |
| Control | 100 |
| Latent TGF-b1 | 170 |
| Latent TGF-b1 | 146 |
| Latent TGF-b1 | 137 |
| PXS64 | 102 |
| PXS64 | 86 |
| PXS64 | 140 |
| Latent TGF-b1+PXS64 | 136 |
| Latent TGF-b1+PXS64 | 103 |
| Latent TGF-b1+PXS64 | 135 |
| Control | 100 |
| --- | --- |
| Control | 100 |
| Control | 100 |
| Latent TGF-b1 | 143 |
| Latent TGF-b1 | 168 |
| Latent TGF-b1 | 147 |
| PXS64 | 107 |
| PXS64 | 102 |
| PXS64 | 122 |
| Latent TGF-b1+PXS64 | 135 |
| Latent TGF-b1+PXS64 | 137 |
| Latent TGF-b1+PXS64 | 84 |

## Slide 4
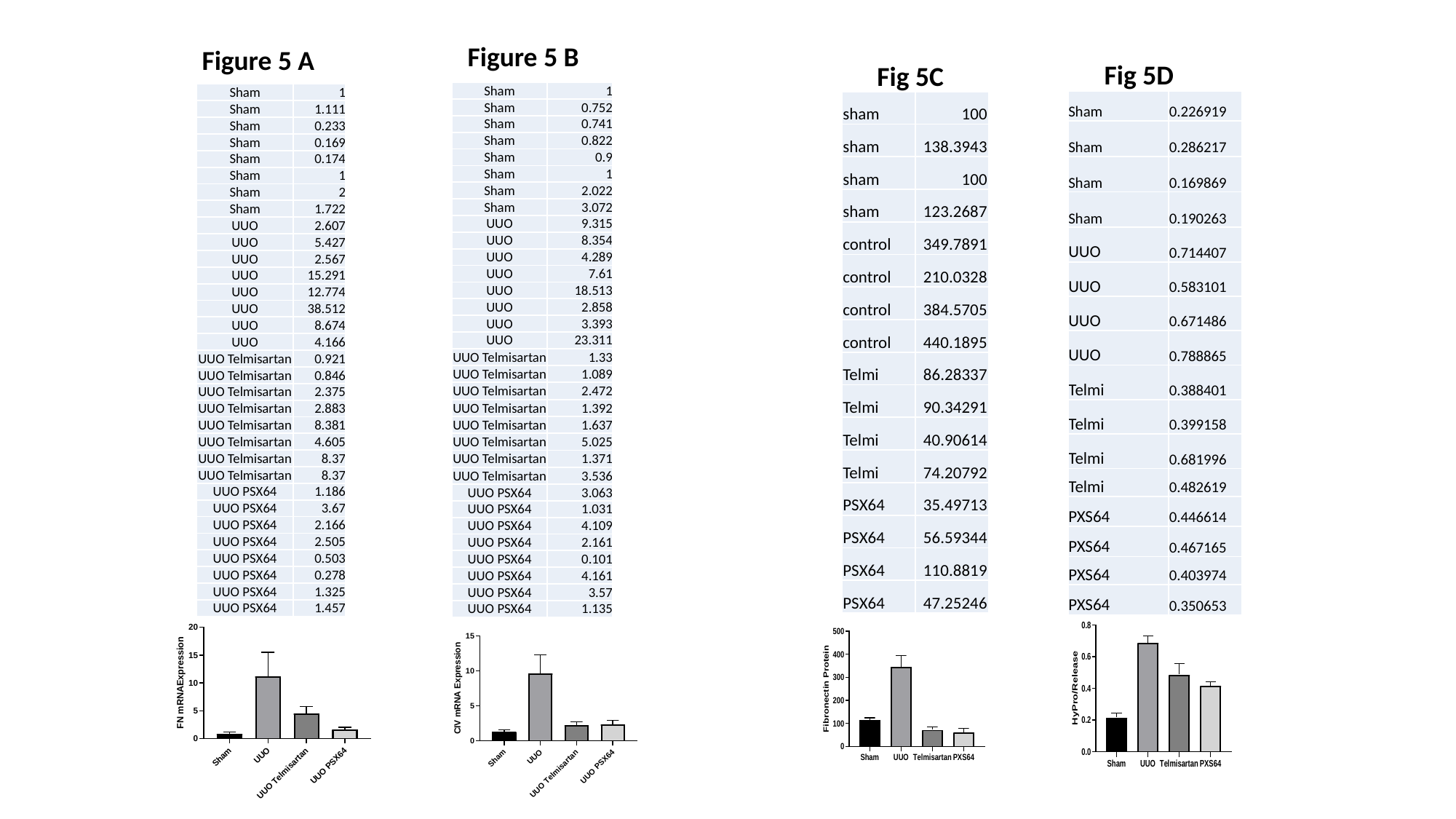

Figure 5 B
Figure 5 A
Fig 5D
Fig 5C
| Sham | 1 |
| --- | --- |
| Sham | 0.752 |
| Sham | 0.741 |
| Sham | 0.822 |
| Sham | 0.9 |
| Sham | 1 |
| Sham | 2.022 |
| Sham | 3.072 |
| UUO | 9.315 |
| UUO | 8.354 |
| UUO | 4.289 |
| UUO | 7.61 |
| UUO | 18.513 |
| UUO | 2.858 |
| UUO | 3.393 |
| UUO | 23.311 |
| UUO Telmisartan | 1.33 |
| UUO Telmisartan | 1.089 |
| UUO Telmisartan | 2.472 |
| UUO Telmisartan | 1.392 |
| UUO Telmisartan | 1.637 |
| UUO Telmisartan | 5.025 |
| UUO Telmisartan | 1.371 |
| UUO Telmisartan | 3.536 |
| UUO PSX64 | 3.063 |
| UUO PSX64 | 1.031 |
| UUO PSX64 | 4.109 |
| UUO PSX64 | 2.161 |
| UUO PSX64 | 0.101 |
| UUO PSX64 | 4.161 |
| UUO PSX64 | 3.57 |
| UUO PSX64 | 1.135 |
| Sham | 1 |
| --- | --- |
| Sham | 1.111 |
| Sham | 0.233 |
| Sham | 0.169 |
| Sham | 0.174 |
| Sham | 1 |
| Sham | 2 |
| Sham | 1.722 |
| UUO | 2.607 |
| UUO | 5.427 |
| UUO | 2.567 |
| UUO | 15.291 |
| UUO | 12.774 |
| UUO | 38.512 |
| UUO | 8.674 |
| UUO | 4.166 |
| UUO Telmisartan | 0.921 |
| UUO Telmisartan | 0.846 |
| UUO Telmisartan | 2.375 |
| UUO Telmisartan | 2.883 |
| UUO Telmisartan | 8.381 |
| UUO Telmisartan | 4.605 |
| UUO Telmisartan | 8.37 |
| UUO Telmisartan | 8.37 |
| UUO PSX64 | 1.186 |
| UUO PSX64 | 3.67 |
| UUO PSX64 | 2.166 |
| UUO PSX64 | 2.505 |
| UUO PSX64 | 0.503 |
| UUO PSX64 | 0.278 |
| UUO PSX64 | 1.325 |
| UUO PSX64 | 1.457 |
| Sham | 0.226919 |
| --- | --- |
| Sham | 0.286217 |
| Sham | 0.169869 |
| Sham | 0.190263 |
| UUO | 0.714407 |
| UUO | 0.583101 |
| UUO | 0.671486 |
| UUO | 0.788865 |
| Telmi | 0.388401 |
| Telmi | 0.399158 |
| Telmi | 0.681996 |
| Telmi | 0.482619 |
| PXS64 | 0.446614 |
| PXS64 | 0.467165 |
| PXS64 | 0.403974 |
| PXS64 | 0.350653 |
| sham | 100 |
| --- | --- |
| sham | 138.3943 |
| sham | 100 |
| sham | 123.2687 |
| control | 349.7891 |
| control | 210.0328 |
| control | 384.5705 |
| control | 440.1895 |
| Telmi | 86.28337 |
| Telmi | 90.34291 |
| Telmi | 40.90614 |
| Telmi | 74.20792 |
| PSX64 | 35.49713 |
| PSX64 | 56.59344 |
| PSX64 | 110.8819 |
| PSX64 | 47.25246 |

## Slide 5
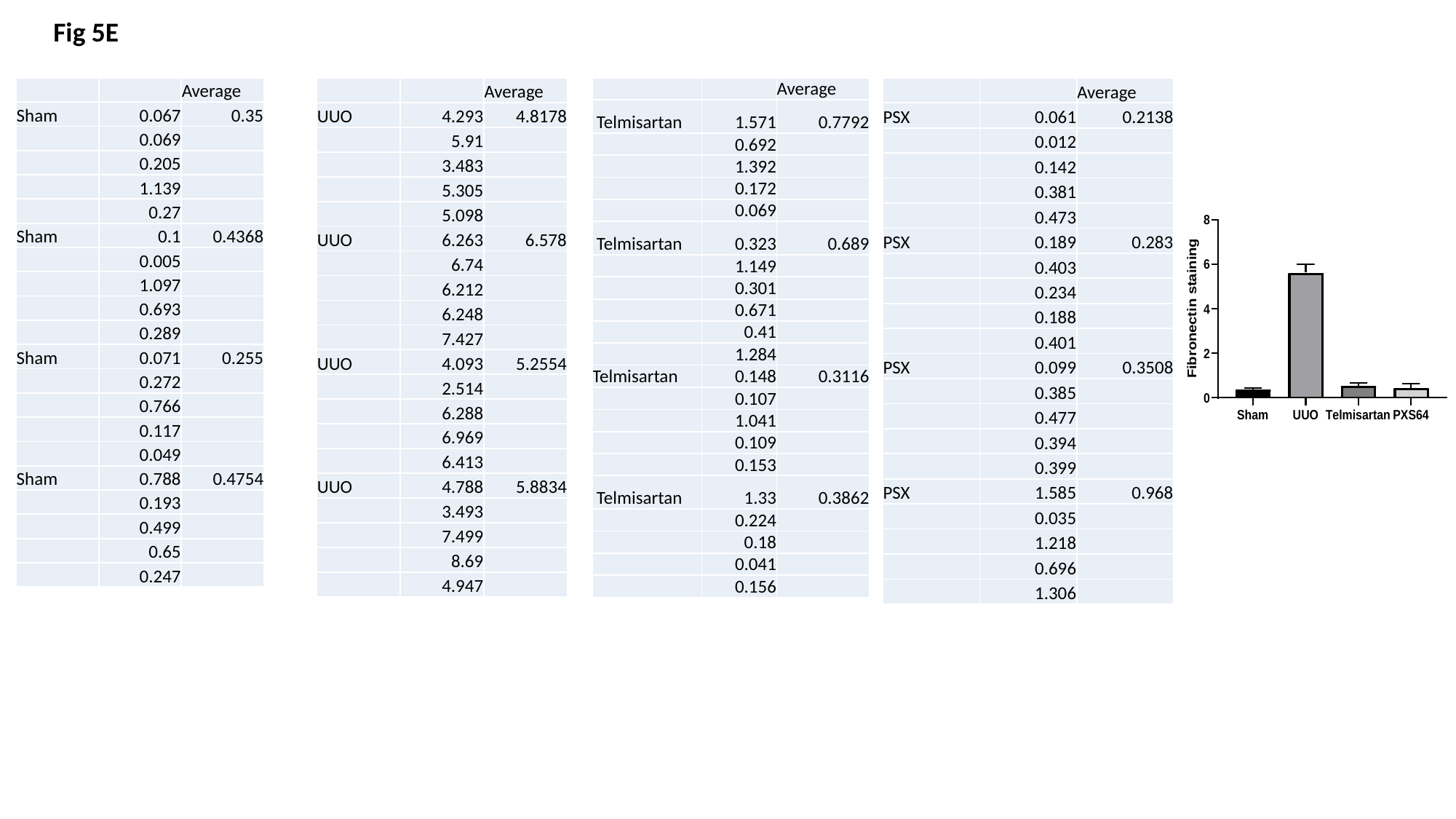

Fig 5E
| | | Average |
| --- | --- | --- |
| UUO | 4.293 | 4.8178 |
| | 5.91 | |
| | 3.483 | |
| | 5.305 | |
| | 5.098 | |
| UUO | 6.263 | 6.578 |
| | 6.74 | |
| | 6.212 | |
| | 6.248 | |
| | 7.427 | |
| UUO | 4.093 | 5.2554 |
| | 2.514 | |
| | 6.288 | |
| | 6.969 | |
| | 6.413 | |
| UUO | 4.788 | 5.8834 |
| | 3.493 | |
| | 7.499 | |
| | 8.69 | |
| | 4.947 | |
| | | Average |
| --- | --- | --- |
| Sham | 0.067 | 0.35 |
| | 0.069 | |
| | 0.205 | |
| | 1.139 | |
| | 0.27 | |
| Sham | 0.1 | 0.4368 |
| | 0.005 | |
| | 1.097 | |
| | 0.693 | |
| | 0.289 | |
| Sham | 0.071 | 0.255 |
| | 0.272 | |
| | 0.766 | |
| | 0.117 | |
| | 0.049 | |
| Sham | 0.788 | 0.4754 |
| | 0.193 | |
| | 0.499 | |
| | 0.65 | |
| | 0.247 | |
| | | Average |
| --- | --- | --- |
| Telmisartan | 1.571 | 0.7792 |
| | 0.692 | |
| | 1.392 | |
| | 0.172 | |
| | 0.069 | |
| Telmisartan | 0.323 | 0.689 |
| | 1.149 | |
| | 0.301 | |
| | 0.671 | |
| | 0.41 | |
| | 1.284 | |
| Telmisartan | 0.148 | 0.3116 |
| | 0.107 | |
| | 1.041 | |
| | 0.109 | |
| | 0.153 | |
| Telmisartan | 1.33 | 0.3862 |
| | 0.224 | |
| | 0.18 | |
| | 0.041 | |
| | 0.156 | |
| | | Average |
| --- | --- | --- |
| PSX | 0.061 | 0.2138 |
| | 0.012 | |
| | 0.142 | |
| | 0.381 | |
| | 0.473 | |
| PSX | 0.189 | 0.283 |
| | 0.403 | |
| | 0.234 | |
| | 0.188 | |
| | 0.401 | |
| PSX | 0.099 | 0.3508 |
| | 0.385 | |
| | 0.477 | |
| | 0.394 | |
| | 0.399 | |
| PSX | 1.585 | 0.968 |
| | 0.035 | |
| | 1.218 | |
| | 0.696 | |
| | 1.306 | |

## Slide 6
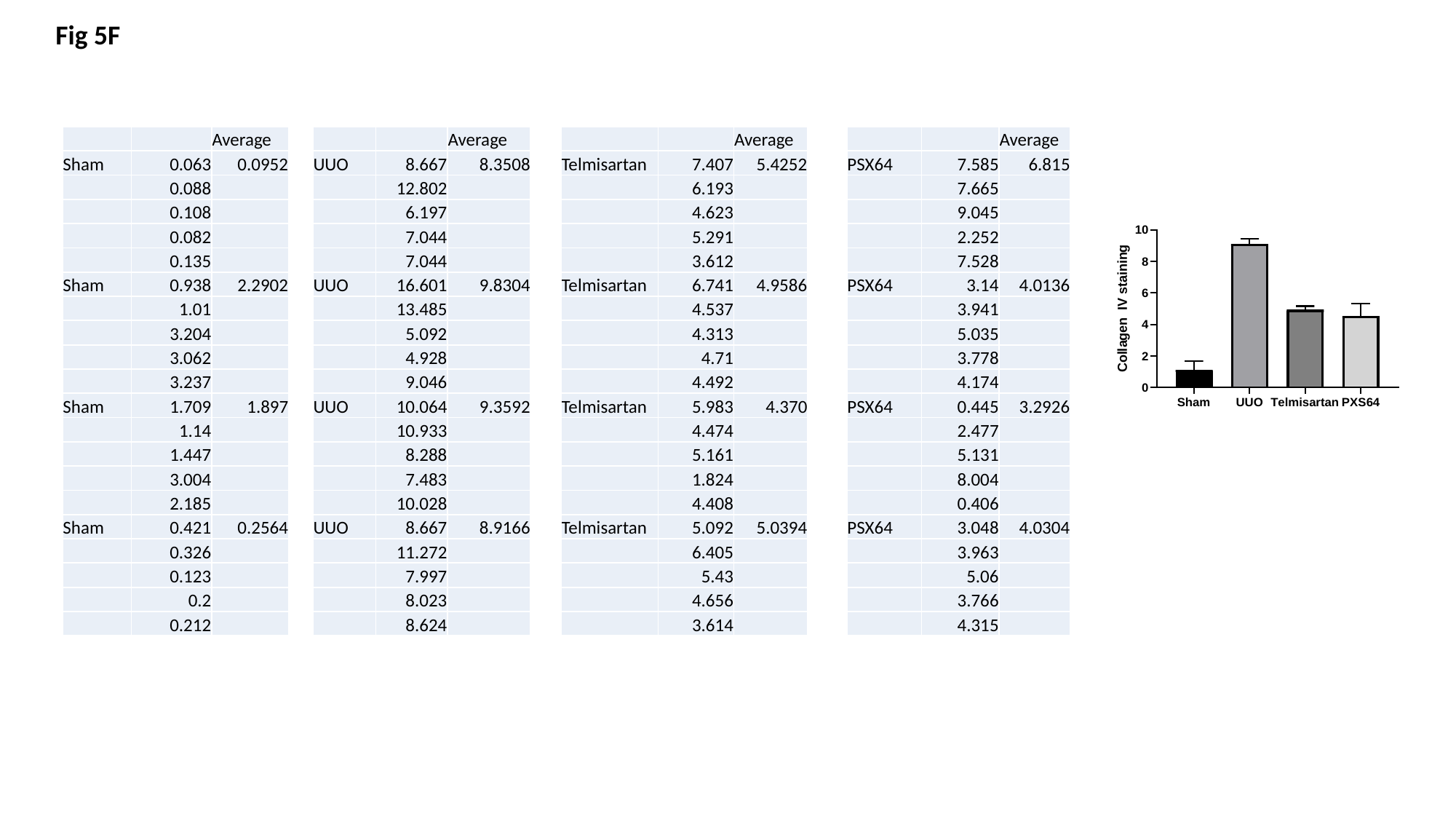

Fig 5F
| | | Average |
| --- | --- | --- |
| Sham | 0.063 | 0.0952 |
| | 0.088 | |
| | 0.108 | |
| | 0.082 | |
| | 0.135 | |
| Sham | 0.938 | 2.2902 |
| | 1.01 | |
| | 3.204 | |
| | 3.062 | |
| | 3.237 | |
| Sham | 1.709 | 1.897 |
| | 1.14 | |
| | 1.447 | |
| | 3.004 | |
| | 2.185 | |
| Sham | 0.421 | 0.2564 |
| | 0.326 | |
| | 0.123 | |
| | 0.2 | |
| | 0.212 | |
| | | Average |
| --- | --- | --- |
| UUO | 8.667 | 8.3508 |
| | 12.802 | |
| | 6.197 | |
| | 7.044 | |
| | 7.044 | |
| UUO | 16.601 | 9.8304 |
| | 13.485 | |
| | 5.092 | |
| | 4.928 | |
| | 9.046 | |
| UUO | 10.064 | 9.3592 |
| | 10.933 | |
| | 8.288 | |
| | 7.483 | |
| | 10.028 | |
| UUO | 8.667 | 8.9166 |
| | 11.272 | |
| | 7.997 | |
| | 8.023 | |
| | 8.624 | |
| | | Average |
| --- | --- | --- |
| Telmisartan | 7.407 | 5.4252 |
| | 6.193 | |
| | 4.623 | |
| | 5.291 | |
| | 3.612 | |
| Telmisartan | 6.741 | 4.9586 |
| | 4.537 | |
| | 4.313 | |
| | 4.71 | |
| | 4.492 | |
| Telmisartan | 5.983 | 4.370 |
| | 4.474 | |
| | 5.161 | |
| | 1.824 | |
| | 4.408 | |
| Telmisartan | 5.092 | 5.0394 |
| | 6.405 | |
| | 5.43 | |
| | 4.656 | |
| | 3.614 | |
| | | Average |
| --- | --- | --- |
| PSX64 | 7.585 | 6.815 |
| | 7.665 | |
| | 9.045 | |
| | 2.252 | |
| | 7.528 | |
| PSX64 | 3.14 | 4.0136 |
| | 3.941 | |
| | 5.035 | |
| | 3.778 | |
| | 4.174 | |
| PSX64 | 0.445 | 3.2926 |
| | 2.477 | |
| | 5.131 | |
| | 8.004 | |
| | 0.406 | |
| PSX64 | 3.048 | 4.0304 |
| | 3.963 | |
| | 5.06 | |
| | 3.766 | |
| | 4.315 | |

## Slide 7
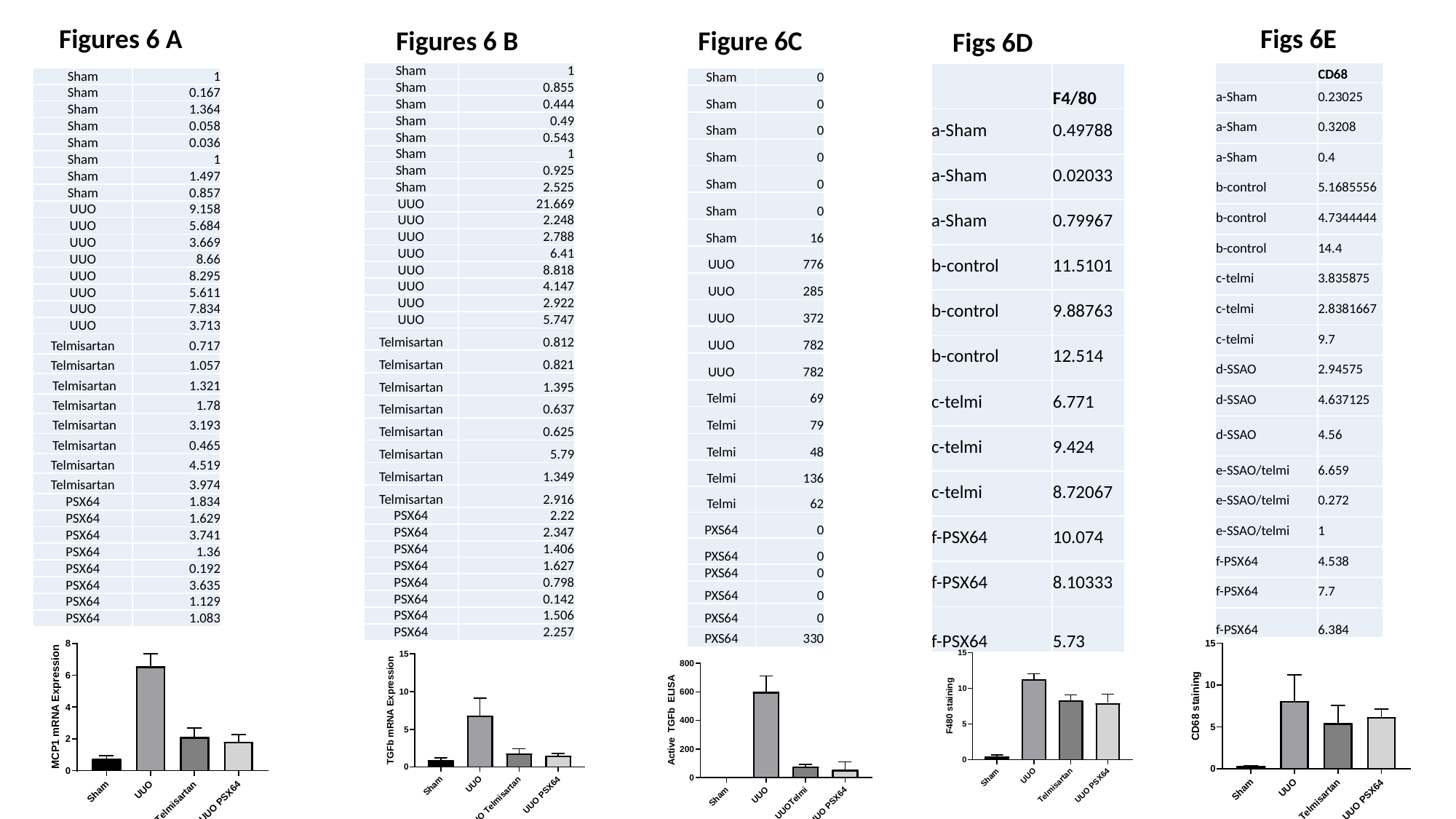

Figures 6 A
Figs 6E
Figures 6 B
Figure 6C
Figs 6D
| Sham | 1 |
| --- | --- |
| Sham | 0.855 |
| Sham | 0.444 |
| Sham | 0.49 |
| Sham | 0.543 |
| Sham | 1 |
| Sham | 0.925 |
| Sham | 2.525 |
| UUO | 21.669 |
| UUO | 2.248 |
| UUO | 2.788 |
| UUO | 6.41 |
| UUO | 8.818 |
| UUO | 4.147 |
| UUO | 2.922 |
| UUO | 5.747 |
| Telmisartan | 0.812 |
| Telmisartan | 0.821 |
| Telmisartan | 1.395 |
| Telmisartan | 0.637 |
| Telmisartan | 0.625 |
| Telmisartan | 5.79 |
| Telmisartan | 1.349 |
| Telmisartan | 2.916 |
| PSX64 | 2.22 |
| PSX64 | 2.347 |
| PSX64 | 1.406 |
| PSX64 | 1.627 |
| PSX64 | 0.798 |
| PSX64 | 0.142 |
| PSX64 | 1.506 |
| PSX64 | 2.257 |
| | CD68 |
| --- | --- |
| a-Sham | 0.23025 |
| a-Sham | 0.3208 |
| a-Sham | 0.4 |
| b-control | 5.1685556 |
| b-control | 4.7344444 |
| b-control | 14.4 |
| c-telmi | 3.835875 |
| c-telmi | 2.8381667 |
| c-telmi | 9.7 |
| d-SSAO | 2.94575 |
| d-SSAO | 4.637125 |
| d-SSAO | 4.56 |
| e-SSAO/telmi | 6.659 |
| e-SSAO/telmi | 0.272 |
| e-SSAO/telmi | 1 |
| f-PSX64 | 4.538 |
| f-PSX64 | 7.7 |
| f-PSX64 | 6.384 |
| | F4/80 |
| --- | --- |
| a-Sham | 0.49788 |
| a-Sham | 0.02033 |
| a-Sham | 0.79967 |
| b-control | 11.5101 |
| b-control | 9.88763 |
| b-control | 12.514 |
| c-telmi | 6.771 |
| c-telmi | 9.424 |
| c-telmi | 8.72067 |
| f-PSX64 | 10.074 |
| f-PSX64 | 8.10333 |
| f-PSX64 | 5.73 |
| Sham | 1 |
| --- | --- |
| Sham | 0.167 |
| Sham | 1.364 |
| Sham | 0.058 |
| Sham | 0.036 |
| Sham | 1 |
| Sham | 1.497 |
| Sham | 0.857 |
| UUO | 9.158 |
| UUO | 5.684 |
| UUO | 3.669 |
| UUO | 8.66 |
| UUO | 8.295 |
| UUO | 5.611 |
| UUO | 7.834 |
| UUO | 3.713 |
| Telmisartan | 0.717 |
| Telmisartan | 1.057 |
| Telmisartan | 1.321 |
| Telmisartan | 1.78 |
| Telmisartan | 3.193 |
| Telmisartan | 0.465 |
| Telmisartan | 4.519 |
| Telmisartan | 3.974 |
| PSX64 | 1.834 |
| PSX64 | 1.629 |
| PSX64 | 3.741 |
| PSX64 | 1.36 |
| PSX64 | 0.192 |
| PSX64 | 3.635 |
| PSX64 | 1.129 |
| PSX64 | 1.083 |
| Sham | 0 |
| --- | --- |
| Sham | 0 |
| Sham | 0 |
| Sham | 0 |
| Sham | 0 |
| Sham | 0 |
| Sham | 16 |
| UUO | 776 |
| UUO | 285 |
| UUO | 372 |
| UUO | 782 |
| UUO | 782 |
| Telmi | 69 |
| Telmi | 79 |
| Telmi | 48 |
| Telmi | 136 |
| Telmi | 62 |
| PXS64 | 0 |
| PXS64 | 0 |
| PXS64 | 0 |
| PXS64 | 0 |
| PXS64 | 0 |
| PXS64 | 330 |

## Slide 8
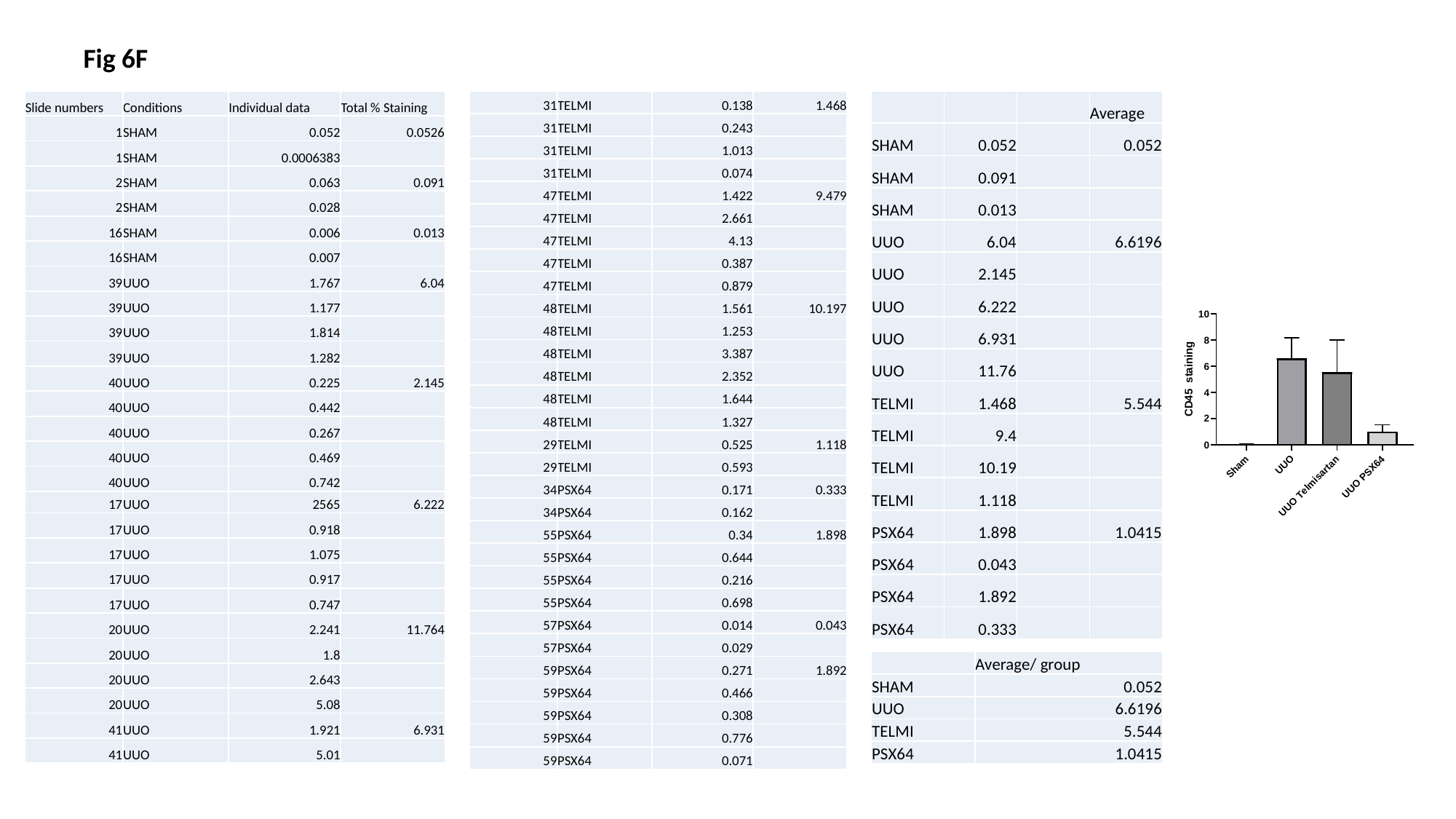

Fig 6F
| | | | Average |
| --- | --- | --- | --- |
| SHAM | 0.052 | | 0.052 |
| SHAM | 0.091 | | |
| SHAM | 0.013 | | |
| UUO | 6.04 | | 6.6196 |
| UUO | 2.145 | | |
| UUO | 6.222 | | |
| UUO | 6.931 | | |
| UUO | 11.76 | | |
| TELMI | 1.468 | | 5.544 |
| TELMI | 9.4 | | |
| TELMI | 10.19 | | |
| TELMI | 1.118 | | |
| PSX64 | 1.898 | | 1.0415 |
| PSX64 | 0.043 | | |
| PSX64 | 1.892 | | |
| PSX64 | 0.333 | | |
| Slide numbers | Conditions | Individual data | Total % Staining |
| --- | --- | --- | --- |
| 1 | SHAM | 0.052 | 0.0526 |
| 1 | SHAM | 0.0006383 | |
| 2 | SHAM | 0.063 | 0.091 |
| 2 | SHAM | 0.028 | |
| 16 | SHAM | 0.006 | 0.013 |
| 16 | SHAM | 0.007 | |
| 39 | UUO | 1.767 | 6.04 |
| 39 | UUO | 1.177 | |
| 39 | UUO | 1.814 | |
| 39 | UUO | 1.282 | |
| 40 | UUO | 0.225 | 2.145 |
| 40 | UUO | 0.442 | |
| 40 | UUO | 0.267 | |
| 40 | UUO | 0.469 | |
| 40 | UUO | 0.742 | |
| 17 | UUO | 2565 | 6.222 |
| 17 | UUO | 0.918 | |
| 17 | UUO | 1.075 | |
| 17 | UUO | 0.917 | |
| 17 | UUO | 0.747 | |
| 20 | UUO | 2.241 | 11.764 |
| 20 | UUO | 1.8 | |
| 20 | UUO | 2.643 | |
| 20 | UUO | 5.08 | |
| 41 | UUO | 1.921 | 6.931 |
| 41 | UUO | 5.01 | |
| 31 | TELMI | 0.138 | 1.468 |
| --- | --- | --- | --- |
| 31 | TELMI | 0.243 | |
| 31 | TELMI | 1.013 | |
| 31 | TELMI | 0.074 | |
| 47 | TELMI | 1.422 | 9.479 |
| 47 | TELMI | 2.661 | |
| 47 | TELMI | 4.13 | |
| 47 | TELMI | 0.387 | |
| 47 | TELMI | 0.879 | |
| 48 | TELMI | 1.561 | 10.197 |
| 48 | TELMI | 1.253 | |
| 48 | TELMI | 3.387 | |
| 48 | TELMI | 2.352 | |
| 48 | TELMI | 1.644 | |
| 48 | TELMI | 1.327 | |
| 29 | TELMI | 0.525 | 1.118 |
| 29 | TELMI | 0.593 | |
| 34 | PSX64 | 0.171 | 0.333 |
| 34 | PSX64 | 0.162 | |
| 55 | PSX64 | 0.34 | 1.898 |
| 55 | PSX64 | 0.644 | |
| 55 | PSX64 | 0.216 | |
| 55 | PSX64 | 0.698 | |
| 57 | PSX64 | 0.014 | 0.043 |
| 57 | PSX64 | 0.029 | |
| 59 | PSX64 | 0.271 | 1.892 |
| 59 | PSX64 | 0.466 | |
| 59 | PSX64 | 0.308 | |
| 59 | PSX64 | 0.776 | |
| 59 | PSX64 | 0.071 | |
| | Average/ group |
| --- | --- |
| SHAM | 0.052 |
| UUO | 6.6196 |
| TELMI | 5.544 |
| PSX64 | 1.0415 |

## Slide 9
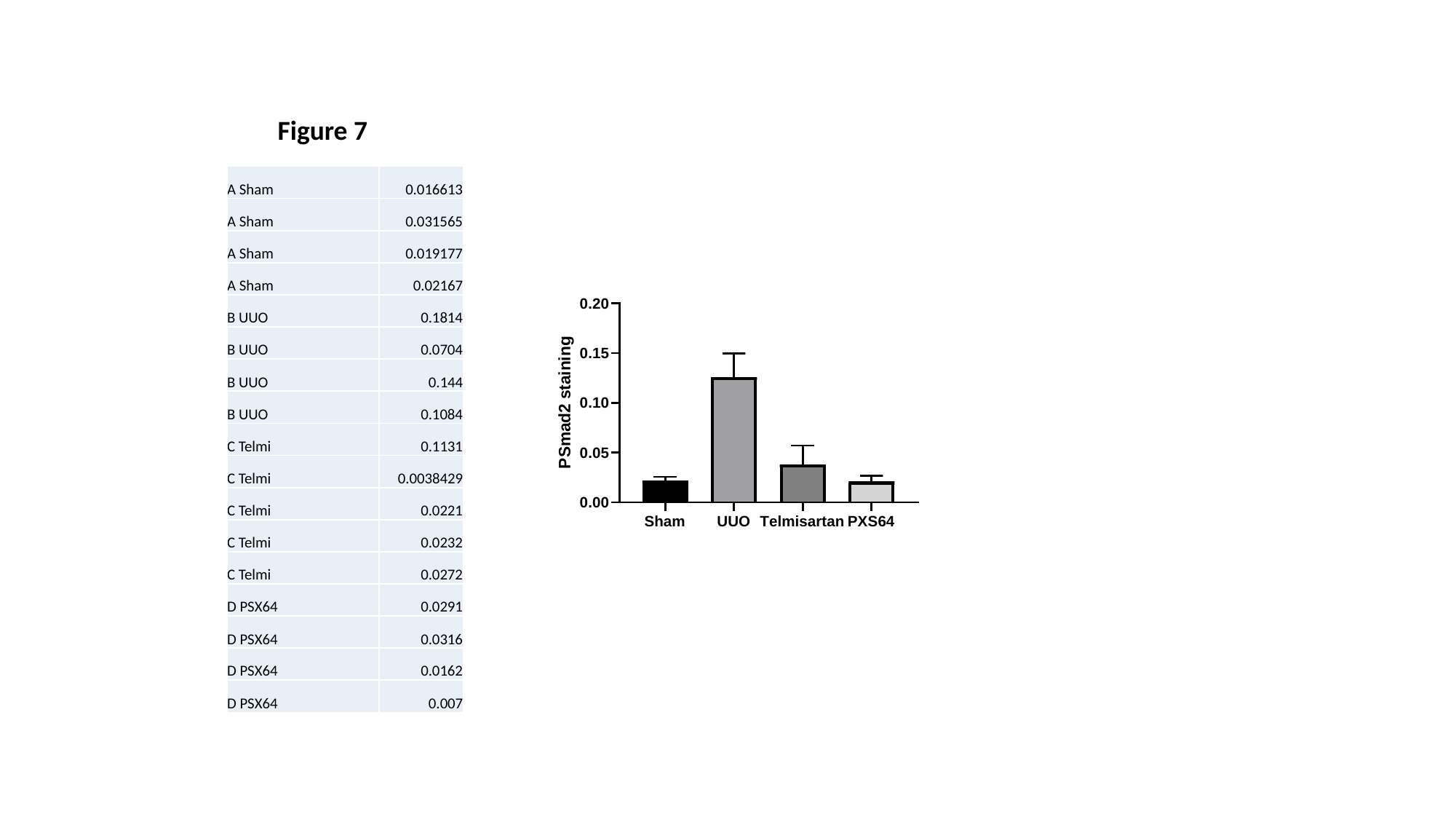

Figure 7
| A Sham | 0.016613 |
| --- | --- |
| A Sham | 0.031565 |
| A Sham | 0.019177 |
| A Sham | 0.02167 |
| B UUO | 0.1814 |
| B UUO | 0.0704 |
| B UUO | 0.144 |
| B UUO | 0.1084 |
| C Telmi | 0.1131 |
| C Telmi | 0.0038429 |
| C Telmi | 0.0221 |
| C Telmi | 0.0232 |
| C Telmi | 0.0272 |
| D PSX64 | 0.0291 |
| D PSX64 | 0.0316 |
| D PSX64 | 0.0162 |
| D PSX64 | 0.007 |
