# Supplementary material for: Correction: A Cationic-Independent Mannose 6-Phosphate Receptor Inhibitor (PXS64) Ameliorates Kidney Fibrosis by Inhibiting Activation of Transforming Growth Factor-β1
Source: PLoS One. 2022 Jan 12;17(1):e0262725. doi: 10.1371/journal.pone.0262725 (PMC8754332; doi:10.1371/journal.pone.0262725)

Figure 4- TIF score

ANOVA Table for Column 2

	DF	Sum of Squares	Mean Square	F-Value	P-Value	Lambda	Pow er
Column 1	5	46.814	9.363	50.269	<.0001	251.345	1.000
Residual	39	7.264	.186				

Means Table for Column 2
Effect: Column 1

	Count	Mean	Std. Dev.	Std. Err.
A-Sham	8	.250	.378	.134
B-Control UUO	8	3.250	.463	.164
C-Telmisartan	8	2.875	.354	.125
PSX64	6	2.500	.548	.224

Fisher's PLSD for Column 2
Effect: Column 1
Significance Level: 5 %

	Mean Diff.	Crit. Diff	P-Value	
A-Sham, B-Control UUO	-3.000	.436	<.0001	S
A-Sham, C-Telmisartan	-2.625	.436	<.0001	S
A-Sham, PSX64	-2.250	.471	<.0001	S
B-Control UUO, C-Telmisartan	.375	.436	.0901	
B-Control UUO, PSX64	.750	.471	.0026	S
C-Telmisartan, PSX64	.375	.471	.1157	

Interaction Bar Plot for Column 2
Effect: Column 1
Error Bars: ± 1 Standard Error(s)

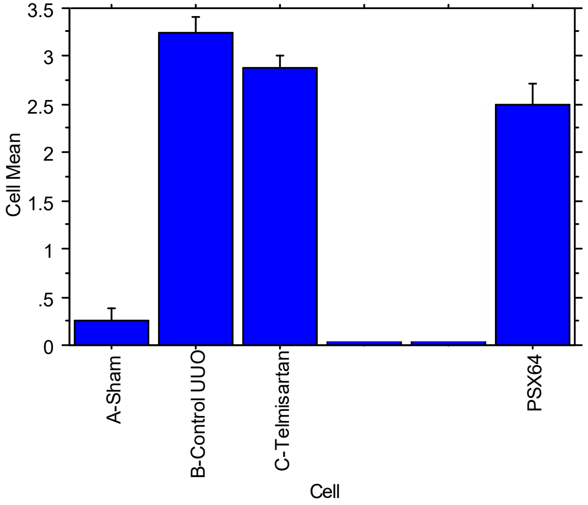

Raw data

Sham	UUO	UUO Telmisartan	UUO PXS64
0.5	3	3	2
0.5	4	2	3
0	3	3	2
0	3	3	2
0	3	3	3
1	4	3	3
0	3	3	
0	3	3	

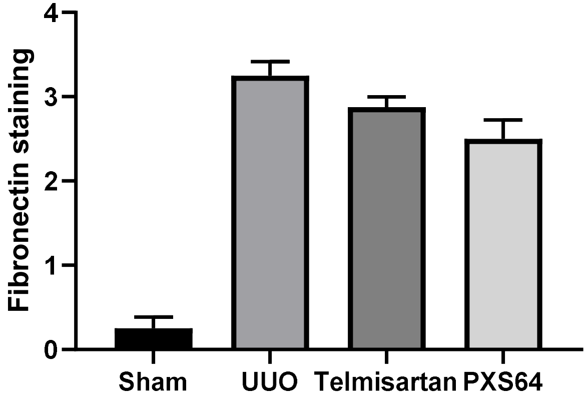

Supplement: S2 File — (PDF) [file pone.0262725.s002.pdf]
